# Antioxidant, Anti-inflammatory and Neuroprotective Profiles of Novel 1,4-Dihydropyridine Derivatives for the Treatment of Alzheimer’s Disease

**DOI:** 10.3390/antiox9080650

**Published:** 2020-07-22

**Authors:** Patrycja Michalska, Paloma Mayo, Cristina Fernández-Mendívil, Giammarco Tenti, Pablo Duarte, Izaskun Buendia, María Teresa Ramos, Manuela G. López, J. Carlos Menéndez, Rafael León

**Affiliations:** 1 Instituto Teófilo Hernando y Departamento de Farmacología y Terapéutica, Facultad de Medicina, Universidad Autónoma de Madrid, 28029 Madrid, Spain; patrycja.michalska@uam.es (P.M.); paloma.mayo@uam.es (P.M.); cristinafm93@gmail.com (C.F.-M.); pablo.duarte@uam.es (P.D.); izaskunbuendia@hotmail.com (I.B.); manuela.garcia@uam.es (M.G.L.); 2 Instituto de Investigación Sanitaria, Servicio de Farmacología Clínica, Hospital Universitario de la Princesa, 28006 Madrid, Spain; 3 Unidad de Química Orgánica y Farmacéutica, Departamento de Química en Ciencias Farmacéuticas, Facultad de Farmacia, Universidad Complutense, 28040 Madrid, Spain; giammarco.tenti@hotmail.it (G.T.); mtramos@farm.ucm.es (M.T.R.); josecm@farm.ucm.es (J.C.M.)

**Keywords:** Alzheimer’s disease, multitarget drugs, 4,7-dihydro-2*H*-pyrazolo[3,4-*b*]pyridines GSK-3β inhibitors, voltage gated calcium channels antagonists, anti-inflammatory drugs, neuroprotection

## Abstract

Alzheimer’s disease is a chronic and irreversible pathological process that has become the most prevalent neurodegenerative disease. Currently, it is considered a multifactorial disease where oxidative stress and chronic neuroinflammation play a crucial role in its onset and development. Its characteristic neuronal loss has been related to the formation of neurofibrillary tangles mainly composed by hyperphosphorylated tau protein. Hyperphosphorylation of tau protein is related to the over-activity of GSK-3β, a kinase that participates in several pathological mechanisms including neuroinflammation. Neuronal loss is also related to cytosolic Ca^2+^ homeostasis dysregulation that triggers apoptosis and free radicals production, contributing to oxidative damage and, finally, neuronal death. Under these premises, we have obtained a new family of 4,7-dihydro-2*H*-pyrazolo[3–*b*]pyridines as multitarget directed ligands showing potent antioxidant properties and able to scavenge both oxygen and nitrogen radical species, and also, with anti-inflammatory properties. Further characterization has demonstrated their capacity to inhibit GSK-3β and to block L-type voltage dependent calcium channels. Novel derivatives have also demonstrated an interesting neuroprotective profile on in vitro models of neurodegeneration. Finally, compound **4g** revokes cellular death induced by tau hyperphosphorylation in hippocampal slices by blocking reactive oxygen species (ROS) production. In conclusion, the multitarget profile exhibited by these compounds is a novel therapeutic strategy of potential interest in the search of novel treatments for Alzheimer’s disease.

## 1. Introduction

Alzheimer’s disease (AD) is a chronic neurodegenerative disease characterized by a progressive memory loss and cognitive dysfunction. Currently, 46.8 million people suffer AD and its prevalence is forecasted to increase to 152 million by 2050 [1]. The World Health Organization considers AD as the pandemic of this century, being a major health and socioeconomic problem due to its chronicity and the tremendous burden that it imposes on caregivers. Furthermore, the only available treatments are symptomatic and they are not able to halt the progression of the disease. Thus, there is an urgent medical need to find an effective drug able to delay or stop the advance of the disease.

AD pathophysiology is characterized by a progressive neuronal loss, mainly at the cholinergic system, and two major hallmarks, intracellular neurofibrillary tangles (NFTs) [2], mainly composed of hyperphosphorylated tau protein (τ), and extracellular plaques constituted by amyloid-β peptide (Aβ) [3]. During the past decades, most of the drug development programs have focused on targeting Aβ, which is consistent with the genetic mutations observed in hereditary cases of AD. However, 95% of the cases are sporadic, indicating different initial causes of the disease. The complex etiology of AD and the mechanistic study of sporadic AD cases have pointed to several pathological pathways involved in the onset of the disease, including mitochondrial failure, oxidative stress (OS), chronic neuroinflammation and proteostasis deregulation. More importantly, these pathological modifications are thought to be initiated almost twenty years earlier than the appearance of the first symptoms [4].

Oxidative stress (OS) is defined as the imbalance between pro-oxidant species (reactive oxygen and nitrogen species, ROS and RNS) and antioxidant defences. Complexes I and III of the mitochondrial electron transport chain are the main ROS generators. Under normal conditions, ROS are key signalling mediators in redox homeostasis, cell death, cell senescence, cellular proliferation, synaptic plasticity and cognitive enhancement, and they can trigger an immune response [5,6]. Under pathological conditions, free radicals can oxidize lipids, DNA and proteins leading to DNA mutations, protein loss of function and, finally, cell death [7]. During aging, the main risk factor of AD, there is a chronic OS imbalance, energy deprivation and decline of antioxidant defense systems that, under certain conditions, have been pointed as potential triggers of the disease [5]. In this line, mitochondrial dysfunction has been described in AD brains [8], together with extensive OS damage and decreased activity of mitochondrial complex IV, as demonstrated in postmortem brains of AD patients [9]**.**

Excessive ROS/RNS production during AD development is thought to participate in the accumulation and aggregation processes of Aβ [10]. Moreover, the Aβ peptide induces OS by increasing ROS production, mitochondrial failure and energy depletion before the appearance of Aβ plaques [11], due to its ability to inhibit complex IV [12]. Recent studies have also demonstrated the capacity of Aβ peptides to impair the import of necessary proteins into the mitochondria through coaggregation, inducing mitochondrial failure and increasing ROS production [13]. Similarly, OS plays a key role in tau hyperphosphorylation through activation of p38 and inactivation of calcineurin (a Ser/Thr phosphatase involved in tau de-phosphorylation) by increasing its natural repressor RCAN1 [14]. Increased levels of RCAN1 also induce the expression of glycogen synthase kinase 3β (GSK-3β) the main kinase related to tau hyperphosphorylation in AD [15], thereby accelerating the formation of hyperphosphorylated tau. Thus, OS induces the aberrant processing of proteins that, in turn, induce mitochondrial damage and additional OS, generating a feedback loop that accelerates neuronal death [16,17]. Recently, a good correlation between the accumulation of NFTs [18] and the cognitive decline associated with AD [19] was demonstrated, highlighting the relevance of tau hyperphosphorylation in this process. GSK-3β is also related to Aβ deposition [20], OS, neuroinflammation [21] and apoptosis, participating in the neuronal death observed in AD [22]. Furthermore, GSK-3β knock-down or inhibition avoids Aβ-induced neurodegeneration [23]. GSK-3β related pathological links in AD include the negative regulation of the most important antioxidant natural defense of cells, the Nrf2-ARE pathway [24]. GSK-3β directly phosphorylates Nrf2, increasing the affinity of the Cul1 adaptor β-TrCP that induces Nrf2 proteasomal degradation [25], thus inhibiting the phase II antioxidant response and contributing to increase OS status. Therefore, renewed interest in GSK-3β as a target has emerged in the context of the multitarget approach by combining it with other emerging targets [26,27,28,29].

Increased OS at early disease stages also initiates the immune response, mainly mediated by glial cells (microglia and astrocytes), by directly acting on scavenger receptors or by the formation of oxidized products that are recognized by other receptors. Activated glia increases the expression of pro-inflammatory enzymes, such as inducible nitric oxide synthase (iNOS) and NADPH oxidase (NOX), among others, increasing the production of RNS and ROS. Nitric oxide (NO) produced by iNOS readily reacts with ROS to produce peroxynitrite, an intermediate that is able to react with Aβ at Tyr10 to increase its aggregation capacity and neurotoxicity [30]. Importantly, nitrated Aβ is the main component found in the core of senile plaques found in the brain of AD patients [31]. Furthermore, Aβ activates the cell surface receptor CD36, inducing an acute immune response that elicits the toll-like receptor 4–6 (TLR4–TLR6) heterodimer that activates the nuclear factor-κB (NF-κB) [32]. In addition, CD36 stimulates Aβ aggregation multiplying its activating capacity [33]. Uncontrolled glial activation induces the excessive expression of cytokines, such as tumor necrosis factor α (TNF-α), interleukin 1β (IL-1β) or IL-18, and chemokines and adhesion molecules to recruit peripheral immune cells [34]. In addition, IL-1β induces neurite destruction and increases P38-MAPK (P38 mitogen-activated protein kinase) kinases [35] and GSK-3β activity, contributing to tau hyperphosphorylation and the appearance of NFTs [36]. Therefore, there is an intense crosstalk between OS and neuroinflammation, starting at the very initial phases of the disease where neither Aβ nor NFT pathologies are initiated [37].

Among the multiple pathological pathways that operate in AD, Ca^2+^ dysregulation has been widely related to the neuronal loss [38,39]. Cytosolic Ca^2+^ ([Ca^2+^]_c_) overload accelerates Aβ production [40], activates GSK-3β [41], increases OS [42] and triggers cellular apoptosis [43]. In turn, Aβ induces [Ca^2+^]_c_ overload leading to ROS production and neuronal death [44]. Recently, it was further demonstrated that selective Ca^2+^ entry through l-type voltage-dependent Ca^2+^ channels (VDCC) increases ROS production and neuronal death [42]. Accordingly, OS toxicity induced by the l-type VDCC Ca^2+^ current was successfully blocked by isradipine, a selective l-type VDCC antagonist [42,45]. In consequence, l-type VDCC antagonists, such as 1,4-dihydropyridines (1,4-DHPs), have been intensively pursued as potential agents to decrease [Ca^2+^]_c_ overload, Aβ production and NFT formation, a combination of targets that may stop the progression of the disease [46].

Based on the complexity and connectivity of the different pathological pathways involved in AD onset and development, it is described as a multifactorial disease [47] whose treatment should be addressed by acting on several targets simultaneously. These reports have boosted the development of novel small molecules designed to act on several targets at the same time, the so-called “multitarget strategy”, to find a neuroprotective drug with disease modifying capacity. Considering the implication of exacerbated OS and neuroinflammation in the onset and development of AD, we report here the synthesis and pharmacological evaluation of a new family of 4,7-dihydro-2*H*-pyrazolo[3,4-*b*]pyridine derivatives, based on the 1,4-DHP core. As mentioned above, 1,4-DHPs are well-characterized selective l-type VDCC blockers with antioxidant and neuroprotective properties [48]. Based on previous results, we have included a pyrazole ring moiety in the structure of new compounds in order to implement GSK-3β inhibitory capacity to decrease tau hyperphosphorylation and, given the relationship of GSK-3β and OS with neuroinflammation, the potential inclusion of anti-inflammatory properties. The obtained derivatives showed potent free radical scavenger capacity, exerted an interesting anti-inflammatory effect, GSK-3β inhibitory capacity at low micromolar range, and moderate VDCC antagonist capacity. Additionally, these compounds showed potent neuroprotective properties against toxicity induced by OS, tau hyperphosphorylation and [Ca^2+^]_c_ overload. Finally, a selected compound reduced cell death in a model of hippocampal slices treated with okadaic acid (OA) by reducing OS.

## 2. Materials and Methods

### 2.1. Synthesis

4,7-Dihydro-2*H*-pyrazolo[3,4-*b*]pyridine derivatives were prepared and purified as described in the Supplementary Material.

### 2.2. Oxygen Radical Absorbance Capacity (ORAC) Assay

ORAC test developed by Cao et al. [49] and modified by Ou et al. [50] was carried out to evaluate the oxygen free radical scavenger capacity of the compounds. (±)-6-Hydroxy-2,5,7,8-tetramethylchromane-2-carboxylic acid (Trolox) (1, 2, 4, 6 and 8 µM) was used as reference compound and melatonin as positive control. Compounds **4a**–**l** were diluted (0.03, 0.1, 0.3, 1, 3 and 10 µM) in phosphate buffered saline (PBS) buffer (10 mM, pH 7.4) at 37 °C and placed a 96-well black microplate (COSTAR 3904). 150 µL of fluorescein (70 nM, final concentration), 25 µL of PBS buffer for blank, 25 µL of Trolox solution for standard and 25 µL of melatonin or compound were added to each well. A fluorescence measurement was done first to determine the basal signal. Then, 25 µL of 2,2′-azobis-amidinopropane dihydrochloride (AAPH) (12 mM, final concentration) were quickly added, since the reaction starts immediately after addition. All samples were carried out in duplicate in three different experiments. Fluorescence was recorded every min for 90 min at 37 °C to obtain the area under the fluorescence decay curve (fluorescence vs time) (AUC) in a FLUOstar Optima plate reader (BMG Labtech, Offenburg, Germany) with 485 nm excitation and 520 nm emission filters. After blank correction and plotting the net AUC vs antioxidant concentration, linear regressions were calculated for all the samples. Final results were expressed in Trolox equivalents (T.eq.), where the ORAC value of Trolox was taken as 1.0.

### 2.3. DPPH Reduction Assay

Experimental conditions were modified from a previously described procedure [51]. Briefly, compounds **4a**–**l** (150 µL) at the desired concentration (1, 10, 60 and 100 µM) in methanol/water (80/20) were added to a solution of 2,2-diphynyl-1-picrylhydrazyl (DPPH) in methanol/water (80/20) (150 µL, 100 µM), in a clear bottom 96-well plate. The final solution was incubated during 1 h in the dark. Then, DPPH absorbance of blank (methanol/water), control (DPPH 100 µM) and compounds plus DPPH were measured at 540 nm in a SPECTROstar Nano plate-reader (BMG Labtech, Ortenberg, Germany) in duplicate. Ascorbic acid and melatonin were used as positive controls. Results are expressed as a percentage of DPPH reduction by subtracting blank absorbance (Abs_blank_) to sample absorbance (Abs_sample_) and corrected by control absorbance, (Abs_control_, DPPH alone, as expressed below:

% DPPH reduction = [100 − ((Abs_sample_ − Abs_blank_) × 100/Abs_control_)] (1)

### 2.4. Mixed Primary Glial Culture

Experimental procedures performed following the Guide for the Care and Use of Laboratory Animals and were previously approved by the Ethics Committee of the Autonomous University of Madrid, Spain (PROEX: 252/16). These procedures were in agreement with the European Guidelines for the use and care of animals for research in accordance with the European Union Directive of 22 September 2010 (2010/63/UE) and with Spanish Royal Decree of 1 February 2013 (53/2013). Mixed glial cultures were obtained from cerebral cortex of 2–5-days-old Sprague-Dawley rats. After the removal of blood vessels and meninges, the forebrains were dissociated mechanically in DMEM/F12 medium. Then, cells were plated (3 × 10^5^ cells/well) in DMEM/F12 medium with 20% fetal bovine serum (FBS) and 1% penicillin/streptomycin (10,000 units), at 37 °C and in a 5% CO_2_-supplemented air atmosphere. After 5 days, medium was substituted by DMEM/F12 medium with 10% FBS. Cells were cultured for 7–10 days before treatment.

### 2.5. Measurement of the Anti-inflammatory Capacity Measured as Nitrite Production Reduction in Mixed Primary Glial Cultures

Cells were pre-incubated with increasing concentrations (0.1, 1, 3, 10 and 30 μM) of compounds for 24 h. Then, treatments were removed and cells were incubated with Lipopolysaccharide (LPS, O26:B6 serotype, 1 μg/mL) in presence of each compound at the desired concentration. Nitrite production was assessed 18 h later by modified Griess assay. Briefly, samples (150 µL) were mixed with 4,4′-diamino-diphenylsulfone (75 µL) and *n*-(1-naphthyl)ethylenediamine (75 µL), and the mixture was incubated at room temperature for 5 min. Light absorption was measured at 550 nm in a microplate reader (Labtech, Offenburg, Germany). All data were normalized to basal nitrite production, considering this value as 100% of nitrite production. EC_50_ values were calculated from dose-response curves represented as percentage of nitrite production reduction induced by the different concentrations of each compound.

### 2.6. GSK-3β Inhibitory Capacity

The method of Baki et al. [52], with some modifications, was followed to analyze the ability of the compounds to inhibit GSK-3β. Tested compounds were dissolved in assay buffer, containing 40 mM Tris (pH 7.5), 20 mM MgCl_2_, 0.1 mg/mL bovine serum albumin (BSA) and 50 µM dithiothreitol (DTT), to achieve final reaction concentrations of 0.1, 1, 10 and 30 µM. Compound SB216763 was used as reference compound. The GSK-3β inhibition assay was performed in white 96-well plates, in duplicates. Firstly, 10 µL of the compounds at the desired concentration were added to each well, followed by 10 µL of enzyme (10 ng). After 30 min of incubation, 20 µL of a mixture of adenosine triphosphate (ATP) and GSK-3β-peptide substrate were added to each well. After a 60 min incubation at 30 °C, the enzymatic reaction was stopped, and the remaining ATP concentration was measured by adding 40 µL/well of Kinase-Glo reagent. IC_50_ values were calculated by non-linear regression analysis of individual concentration-response curves using GraphPad Prism software (San Diego, CA, USA). *Ki* values were calculated using the Chen-Prusoff equation simplified:

*K_i_* = IC_50_/(1 + (S/*K_m_*)) (2)

Being *S* the substrate concentration (ATP) in the experiment, and *K_m_* the Michaelis-Menten constant of the substrate for the enzyme (*K_m_* = 2.345 μM for ATP).

### 2.7. SH-SY5Y Neuroblastoma Cell Line Culture

SH-SY5Y human neuroblastoma cells (obtained from ATCC, Manassas, VA, United States, CRL-2266) were cultured according to supplier directions in a 1:1 mixture of F12 (Ham 12) and Eagle’s Minimum Essential Medium (MEM), supplemented with 15% non-essential amino acids, 10% heat-inactivated FBS, 0.5 mM sodium pyruvate, 24 mM NaHCO_3_, 100 μg/mL streptomycin and 100 units/mL penicillin. Cells were maintained at 37 °C in a humidified atmosphere of 95% air and 5% CO_2_. For experimental procedures, cells were cultured in 96-well plates (6 × 10^4^ cells per well). Treatments were carried out in 1% FBS medium unless other concentration was specified. Cells were used from 4 up to 13 passages.

### 2.8. Voltage Dependent Calcium Channels Blockade Assay

Free cytosolic Ca^2+^ was measured using the fluorescence Ca^2+^ indicator Fluo-4/AM. SH-SY5Y cells were seeded in 96-well black bottom transparent plates at a density of 60,000 cells per well and cultured until they reach confluence. Cells were incubated with the Ca^2+^ sensitive dye Fluo-4/AM at 5 µM in Krebs-HEPES buffer (KH) (145 mM NaCl, 4.9 mM KCl, 1.2 mM MgCl_2_, 2 mM CaCl_2_, 10 mM HEPES, 11 mM glucose, pH 7.4), and 0.05% pluronic acid, for 1 h at 37 °C in the dark. Then, cells were washed twice with KH solution to remove the excess of probe and compounds in solution were added. Test compounds at 10 µM were incubated 10 min before K^+^ 70 mM was applied to trigger cytosolic Ca^2+^ increase. Fluorescence was measured in a fluorescence microplate reader (FLUOstar Optima, BMG, Ortenberg, Baden-Wuerttemberg, Germany) at excitation and emission wavelengths 485 and 520 nm, respectively. To normalize Fluo-4 signals, Triton X-100 (5%) and subsequently MnCl_2_ 1 M were applied to the cells to register maximal and minimal fluorescence, respectively. Data were calculated as a percentage of Fluorescence increment with respect to F_max_ − F_min_. The percentage of response was calculated considering basal conditions as 100% response.

### 2.9. Blood-Brain Barrier Permeation Assay (PAMPA)

Prediction of the capacity of compounds to cross the blood–brain barrier (BBB) by passive diffusion was evaluated using a Parallel Artificial Membrane Permeation Assay (PAMPA), in a similar manner as described previously [53]. The effective permeability of the compounds was measured in duplicate at an initial concentration of 100 µM, including reference positive and negative compounds (see Supporting Information, Table SI1). The compounds of interest were dissolved in 10 mM phosphate-buffered saline (PBS) buffer (pH 7.4) to the desired concentration in the donor well (Multiscreen IP sterile clear plate PDVF membrane, pore size 0.45 μM). The acceptor 96-well plate (Multiscreen, Millipore Corp. Burlington, MA, United States) was filled with 180 µL of PBS (VA). The filter membrane of the donor 96-well plate was impregnated with 4 µL of porcine brain lipid (PBL) (Avanti Polar Lipids, Inc., Alabaster, AL, United States) in dodecane (20 mg/mL) (Sigma-Aldrich, Madrid, Spain), and after 5 min, 180 µL of each compound solution was added to determine their ability to pass the brain barrier (VD). Then, the donor filter plate was carefully put on the acceptor plate to form a “sandwich”, which was left undisturbed for 4 h at 25 °C. After incubation, the donor plate was carefully removed. An ultraviolet (UV) plate reader determined the concentration of compounds and commercial drugs in both the acceptor and the donor wells (150 µL/well) as the maximum absorption wavelength of each compound. Concentration of the compounds in the donor and acceptor well and equilibrium concentration were calculated from the standard curve and expressed as the permeability (Pe), according the equation [1] (see supporting information). Results are expressed as mean ± SEM of three different experiments in duplicate.

### 2.10. Molecular Docking Calculations

GSK-3β structure was prepared and minimized using MacroModel (v2018-1, Schrödinger, Inc., New York, NY, United States) with Optimized Potentials for Liquid Simulations 3 (OPLS3) force field. Next, molecular docking was performed using AutoDock Vina (v1.1.2, Molecular Graphics Lab, Scripps Research Institute, San Diego, CA, United State [54] with center of the box located on the ATP binding site, and the best nine poses were considered. The selected 4,7-dihydro-2*H*-pyrazolo[3,4-*b*]pyridine **4f** (*R*)- and (*S*)- enantiomers were submitted to molecular docking with the previous specifications [28]. The same docking strategy was used for predictions with Ca_V_1.2 L-type channel and selected 4,7-dihydro-2*H*-pyrazolo[3,4-*b*]pyridine compound **4f** in the 1,4-DHP binding site. As a crystal structure of this subtype is not available, we used the Ca_V_1.2 l-subtype VDCC model developed by Denis Tikhonov and Boris S. Zhorov and kindly shared with us by Prof. Zhorov [55].

### 2.11. Neuroprotection Studies in the SH-SY5Y Human Neuroblastoma Cell Line

Cells were pre-incubated with the corresponding compound at 1 μM in neuroblastoma culture medium. After 24 h, the medium was removed and replaced with 1% FBS neuroblastoma culture media containing the corresponding compound at 1 µM and the toxic stimuli, namely a mixture of rotenone and oligomycin A (R/O, 30 μM/10 μM, respectively), okadaic acid (OA) at 20 nM or a high concentration of KCl (70 mM). Cells were co-incubated for further 24 h with the R/O mixture or KCl solution, or 18 h with the OA solution. Melatonin (1 µM) or nimodipine (1 µM) were used as positive control and reference compounds in the R/O, OA or high concentration of potassium models, respectively. Control cells were incubated with the same amount of dimethyl sulfoxide (DMSO) without any drug. After the co-incubation period, cell viability was assessed by the 3-(4,5-dimethylthiazol-2-yl)-2,5-diphenyltetrazolium bromide (MTT) reduction method [56]; basal condition was considered as 100% survival.

### 2.12. Acute Treatment of Rat Hippocampal Slices

The 3–4 month-old Sprague-Dawley rats were decapitated and both hippocampi were dissected and placed in a previously oxygenated (95% O_2_ and 5% CO_2_) ice-cold Krebs’s dissection buffer (120 mM NaCl; 2 mM KCl; 26 mM NaHCO_3_; 1.18 mM KH_2_PO_4_; 10 mM MgSO_4_; 0.5 mM CaCl_2_; 11 mM glucose and 200 mM sucrose at pH 7.4). Hippocampi were cut into 250 µm thick slices using a McIlwain Tissue Chopper (Cavey Laboratory Engineering, Surrey, United Kingdom) and separated using a Leica SE6 microscope (Leica, Madrid, Spain). To allow slice recovery from the previous slicing trauma, they were transferred to a vial containing dissection buffer without sucrose, bubbled with 95% O_2_ and 5% CO_2_ for 45 min at 34 °C (stabilization). Thereafter, the slices were placed in a 48-well plate containing new medium composed of 1:1 control buffer (120 mM NaCl; 2 mM KCl; 26 mM NaHCO_3_; 1.18 mM KH_2_PO_4_; 10 mM MgSO_4_; 2 mM CaCl_2_ and 11 mM glucose) and DMEM/F12 medium (Invitrogen, Madrid, Spain). Slices were treated with saline, OA (1 µM) or OA co-incubated with the corresponding compounds (10 µM), and the plate was maintained under culture conditions (at 37 °C, 5 % CO_2_ and 95 % O_2_) for 6 h. Finally, cell viability and ROS production were measured.

### 2.13. Measurement of ROS Production in Rat Hippocampal Slices

ROS quantification was performed using the fluorescent probe 2´,7´-dichlorodihydrofluorescein diacetate (H_2_DCFDA) (λ_Ex/Em_ = 495/529 nm) (Thermo Fisher, Waltham, MA, USA). Slices were incubated with the probe for 45 min at a concentration of 10 µL/mL, in the presence of 1 µL/mL of Hoechst at 37 °C before finalizing the experiment. Fluorescence from the cornu Ammonis 1 (CA_1_) was measured as mean intensity at an excitation wavelength of 480 nm and an emission of 520 nm for DCFDA in an inverted Nikon Eclipse T2000-U microscope (Nikon, Tokyo, Japan), with a 2X objective. Results were normalized to basal condition, which was considered as 100 %. NIS-Elements BR 4.10.04 64-bit software (NIS BR 4.10.04, Nikon, Tokyo, Japan) was used to analyze the images.

### 2.14. Statistical Analysis

All values are expressed as mean ± S.E.M. IC_50_ and LD_50_ values were calculated by non-linear regression analysis of individual concentration-response curves using GraphPad Prism software (v6.0, GraphPad, San Diego, CA, USA). Analysis of the results was performed by comparison between experimental and control data using one-way ANOVA followed by Newman-Keuls post-hoc test when three groups were implicated. Differences were considered to be statistically significant when *p* ≤ 0.05; “*n*” represents the number of different cultures used or enzyme inhibition assays performed.

## 3. Results and Discussion

### 3.1. Synthesis of 4,7-Dihydro-2H-Pyrazolo[3,4-b]Pyridine Derivatives

**Scheme 1:**
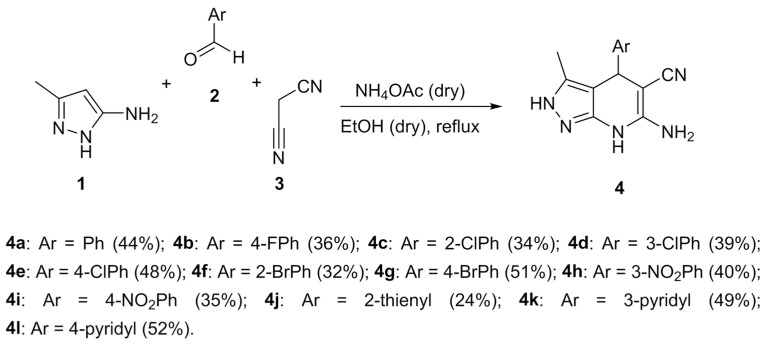
Multicomponent synthetic approach to the 4,7-dihydro-2*H*-pyrazolo[3,4-*b*]pyridine derivatives **4a**–**l**.

The synthesis of the 4,7-dihydro-2*H*-pyrazolo[3,4-*b*]pyridine derivatives **4** was performed by using a Hantzsch-like multicomponent-reaction approach from 3-methyl-1*H*-pyrazol-5-amine **1**, aromatic aldehydes **2a**–**l** and malononitrile **3** [28]. This mixture was refluxed in dry ethanol in the presence of dry ammonium acetate (Scheme 1) to obtain the desired compounds **4a**–**l**.

Under these conditions, we were able to obtain a library of substituted Ph derivatives bearing electron-withdrawing substituents at the aromatic ring (compounds **4a**–**i**), as well as three compounds bearing heterocyclic substituents (**4j**–**l**). Regarding reactions involving aromatic aldehydes with electron-donating groups, we obtained only traces of the corresponding pyrazolo[3,4-*b*]pyridine derivatives from highly complex reaction mixtures, due to full oxidation to the completely aromatic pyridine system.

### 3.2. Pharmacological Evaluation

#### 3.2.1. Antioxidant and Anti-inflammatory Properties of Novel 4,7-Dihydro-2H-Pyrazolo[3,4-b]Pyridine Derivatives **4a**–**l**

As described previously, OS is considered a prominent pathological factor, not only in the progression of AD, but also in the onset and initial phases of the disease. Particularly, 1,4-DHPs are considered a specific type of antioxidants due to their ability to inhibit free radical reactions by acting as hydrogen donors and their capacity to be oxidized under biological conditions [48]. Thus, we tested the potential antioxidant effect of our 4,7-dihydro-2*H*-pyrazolo[3,4-*b*]pyridine derivatives as direct ROS scavengers using the ORAC test, and melatonin, a potent natural antioxidant molecule, as positive control. As reported in Table 1, melatonin successfully scavenged oxygen free radicals being 2.14-fold more potent than trolox. Compounds **4a**–**l** demonstrated scavenger properties showing ORAC values between 0.63 T.eq. (**4d**, Ar = 3-ClPh) and 2.93 T.eq. (**4j**, Ar = 2-thienyl), the latter being more potent than melatonin. Considering phenyl derivatives, only compound **4b** showed higher antioxidant capacity than trolox (1.86 T. eq.) being the most potent of the carbocyclic derivatives. To delve into the antioxidant characterization of compounds **4a**–**l**, we evaluated their potential capacity to scavenge RNS by using the DPPH method. Ascorbic acid and melatonin were included as positive control and reference for comparative purposes. In general, compounds **4a**–**l** showed increased scavenger effect towards RNS compared to melatonin, being in all cases more potent. Furthermore, they showed almost 3-fold higher antioxidant capacity than ascorbic acid, a potent RNS scavenger [57]. RNS scavenger capacity was similar in all cases with IC_50_ values ranging from 17.1 μM (**4k**, Ar = 3-Py) to 25.2 μM (**4a**, Ar = Ph). This result indicates that the scavenger effect is directly related to the 4,7-dihydro-2*H*-pyrazolo[3,4-*b*]pyridine core, as it has been shown for different 1,4-DHPs described previously [58,59].

Neuroinflammation is a widely described pathological pathway present also at the initial phases of AD [60,61]. Furthermore, as depicted in the introduction section, extensive OS induces glial activation by raising the production of pro-inflammatory mediators. These mediators augment ROS and RNS to generate a positive feedback loop leading to chronic neuroinflammation, a highly toxic environment for neurons described as one of the pathological hallmarks of AD. With these premises, we tested the potential anti-inflammatory capacity of compounds **4a**–**l** in primary rat glial cultures stimulated with LPS. TLR4/2 activation by LPS induces iNOS overexpression that increases NO production. Compounds **4a**–**l** were evaluated at increasing concentrations (0.1, 1, 3, 10 and 30 μM) and EC_50_ values were calculated from non-lineal regression of nitrite production reduction. Sulforaphane, a potent anti-inflammatory agent was included as positive control and for comparative purposes. Interestingly, all compounds showed anti-inflammatory capacity with EC_50_ values ranging from 780 nM of compound **4d** (Ar = 3-ClPh) to 22.3 μM of compound **4k** (Ar = 3-Py) (Table 1). With respect to the heterocyclic derivatives, only the 2-thienyl derivative showed an EC_50_ lower than 10 μM.

#### 3.2.2. GSK-3β Inhibition, Voltage-Dependent Ca^2+^ Channels Blockade and BBB Permeation Properties

Among the pro-inflammatory pathways triggered before and after disease onset, GSK-3β is extensively described as an important kinase related to tau hyperphosphorylation and pro-inflammatory signal transduction [62,63]. Thus, we envisaged studying the GSK-3β inhibitory capacity of derivatives **4a**–**l**. To this end, we used the luminescence Kinase-Glo assay at four different concentrations (0.1, 1, 10 and 30 μM). IC_50_ values and their related apparent equilibrium dissociation constants (*K_i_*) are summarized in Table 2, including compound SB216763, a potent GSK-3β inhibitor, as a positive control. Compound **4a** showed an improved inhibitory capacity compared to its pyrano[2,3-*c*]pyrazole isoster [28], indicating an important role of the 1,4-DHP ring in the interaction with GSK-3β. Interestingly, the inclusion of substituents at the aromatic moiety at C-4 position increased their capacity to inhibit GSK-3β, showing IC_50_ values from 820 nM of derivative **4h** (Ar = 3-NO_2_Ph) to 10.2 μM of compound **4l** (Ar = 4-Py). Considering substituent patterns, the tendency indicated that GSK-3β inhibitory potency increased when the substituent was inserted in *ortho* and *meta* positions compared to *para* position. Focusing on heterocyclic derivatives, the addition of an electron-rich heterocycle (thiophene) increased the inhibitory potency, compared to an electron-deficient ring (pyridine), the thienyl derivative being **4j** two-fold more potent than **4k** (Ar = 3-Py) and 5-fold more potent than **4l** (Ar = 4-Py).

As discussed, inflammatory pathway activation is partially mediated by GSK-3β; thus, the inhibitory properties of this kinase by compounds **4a**–**l** might be related to their anti-inflammatory capacity. Indeed, the anti-inflammatory properties of ClPh and NO_2_Ph derivatives correlated with GSK-3β inhibitory capacity, being *meta* derivatives most potent in both assays. Similar conclusions can be extracted considering the heterocyclic substituents. In this case, the most potent GSK-3β inhibitor, compound **4j** (Ar = 2-thienyl, IC_50_ = 1.74 μM) showed the most potent anti-inflammatory properties (EC_50_ = 5.4 μM). However, bromine-substituted derivatives did not show the same tendency, since *o*-BrPh derivative **4f**, with the best IC_50_ towards GSK-3β (IC_50_ = 0.83 μM) is a poor anti-inflammatory compound (EC_50_ = 10.1 μM), while *p*-BrPh derivative **4g** (GSK-3β IC_50_ = 2.35 μM) was 2.2-fold more potent as anti-inflammatory (nitrite reduction EC_50_ = 4.5 μM) than **4f**. These results indicate that the anti-inflammatory properties of compounds **4a**–**l** could be partially dependent on the GSK-3β inhibitory capacity, and that their antioxidant properties participate in its anti-inflammatory mechanism of action.

As previously described, specific Ca^2+^ overload through L-type VDCC has been directly related to OS generation [42,45] and neuronal death. In this regard, the 1,4-DHP ring has specific activity towards L-type VDCCs. Thus, compounds **4a**–**l** were tested as potential VDCC antagonist at 10 μM and the results are shown as percentage of response after membrane depolarization induced by 70 mM K^+^ stimuli in the SH-SY5Y neuronal cell line. Nimodipine was included as positive control at the same concentration. In general, compounds **4a**–**l** showed interesting antagonistic activity reducing Ca^2+^ entrance to 85.6 % (**4k**) up to only 6.46 % response (**4e**). Compounds **4e** and **4f** (Ar = 4-ClPh and Ar = 2-BrPh derivatives, respectively) showed a highly effective blockade of [Ca^2+^]_c_ elevation, being more effective than nimodipine at the same concentration. Interestingly, they contain a halogen substitution, as other 1,4-DHPs used in the clinic as VDCC antagonists that are being tested for mild cognitive impairment [66].

Finally, considering the potential use of our compounds as AD treatment, we explored the BBB penetration using the parallel artificial membrane permeation assay (PAMPA) prediction method. The in vitro permeabilities (*Pe*) of compounds **4a**–**l** were calculated using the lipid extract of porcine brain (Table 2). We have used a range of positive and negative controls (see Supporting Information, Table SI1). The results depicted in Table 2 indicate that, excluding **4h** (Ar = 3-NO_2_Ph), **4i** (Ar = 4-NO_2_Ph) and **4k** (Ar = 3-Py), all compounds are predicted to be able to cross the BBB by passive diffusion with *Pe* values higher than 4, a widely accepted value that marks the limit for passive diffusion [53].

### 3.3. Molecular Docking: Proposed Binding Mode of Compound 4f at the ATP Binding Site of GSK-3β and the 1,4-DHP Binding Site of L-Type VDCC

**Figure 1:**
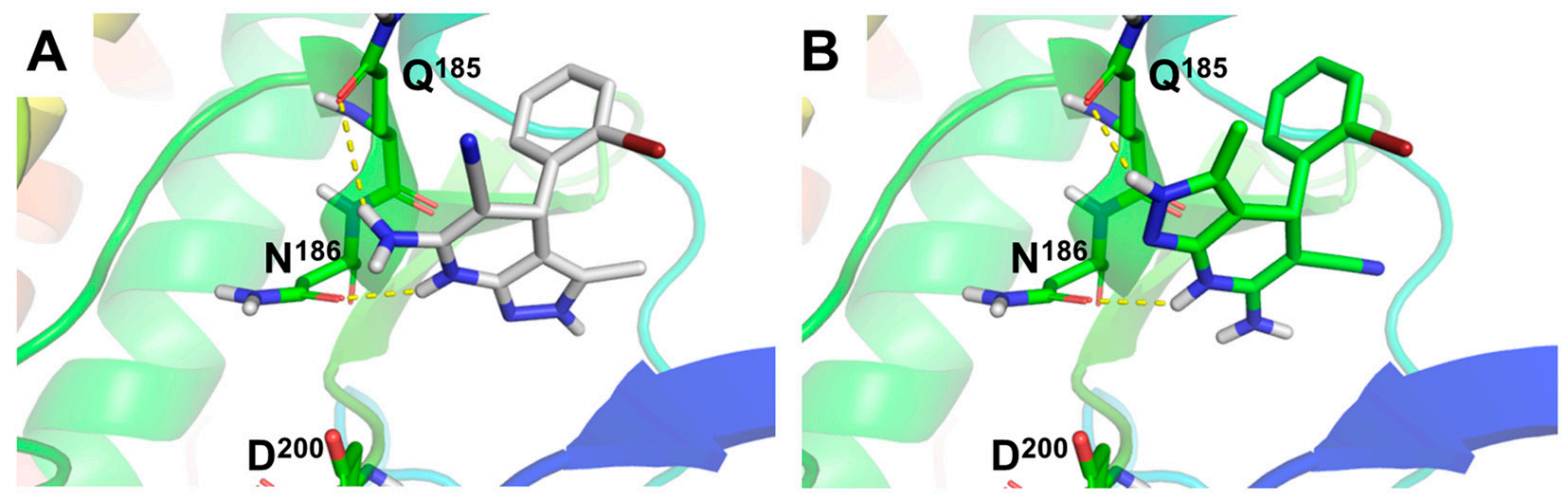
Molecular docking of compound 4f (Ar = 2-BrPh), (*R*)- and (*S*)- enantiomers, at the adenosine triphosphate (ATP) binding site of GSK-3β. (**A**) Predicted binding mode for 4f (*R*)-enantiomer and (**B**) (*S*)-enantiomer represented as white and green sticks, respectively. Hydrogen bond interactions showed as yellow dotted lines. GSK-3β protein (PDB-ID: 3I4B) represented as colored cartoon and key residues as colored sticks.

In order to understand the GSK-3β inhibition ability of compounds **4a**–**l**, we studied the binding mode of the most potent GSK-3β inhibitor, derivative **4f** (Ar = 2-BrPh), to GSK-3β (Figure 1) by means of molecular docking experiments. To this end, we evaluated both enantiomers at the ATP binding site of the enzyme. Results afforded similar poses for the (*R*)- and (*S*)- enantiomers of **4f**, overlapping the pyridine ring with a 180° flip, leaving the pyrazole core on opposite sides. Both enantiomers form stable hydrogen bonds with Q^185^ and N^186^ residues (Figure 1A,B). Remarkably, the hydrogen bond between the NH group of the 1,4-DHP ring of both enantiomers and N^186^ emphasizes the importance of this group for its inhibitory activity. Additionally, both enantiomers showed hydrogen bonding with Q^185^. The (*R*)-enantiomer forms this interaction through the amino group at the 1,4-DHP ring (Figure 1A), while the (*S*)-enantiomer uses the NH moiety at the pyrazole ring (Figure 1B), in line with the predicted 180° flip binding mode shown in Figure 1. Compound **4f** predicted poses were located in the surroundings of D^200^, key residue for ATP stabilization in the substrate cavity (see Figure 1 and Supporting Information, Figure SI1).

**Figure 2:**
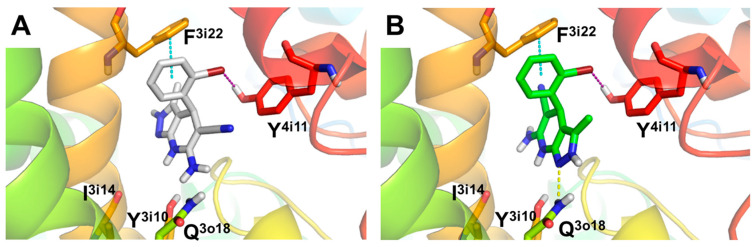
Molecular docking of compound 4f (Ar = 2-BrPh), (*R*)- and (*S*)- enantiomers, at the 1,4-DHP binding site of Ca_V_1.2 l-type VDCC. (**A**) Predicted binding mode for 4f (*R*)-enantiomer and (**B**) (*S*)-enantiomer represented as white and green sticks, respectively. Hydrogen bond, halogen bond and π-π stacking interactions showed as yellow, purple and blue dotted lines, respectively. Ca_V_1.2 L-type channel structure represented as colored cartoon with key residues as sticks.

Considering that compound **4f** showed a potent L-type VDCC blockade capacity (% response = 7.39%), we also carried out molecular docking studies to determine the binding mode and potential interactions with the Ca_V_1.2 L-type VDCC. The L-type VDCC crystal structure is not available; thus, we used a structural homology model of the channel developed by Zhorov et al. [55]. As previously done, we performed docking studies of both enantiomers of **4f** at the 1,4-DHP L-type VDCC binding site located at the interface of domains III and IV of the α1 pore-constituent subunit (Figure 2). Docking results predicted a binding mode similar to that previously described for nimodipine (see Supporting Information, Figure SI2) [55]. The results obtained indicate that compound **4f** places the 1,4-DHP ring of both enantiomers in a similar position to (*S*)-nimodipine, although they are slightly displaced towards the I^3i14^ and Y^3i10^ residues (Figure 2 and Supporting Information, Figure SI2). In this sense, the stern and bowsprit positions of **4f** are close to those of (*S*)-nimodipine (see Supporting Information, Figure SI2). Both enantiomers showed a similar pose with a 180° flip generating similar interactions with key residues known to be involved in 1,4-DHPs stabilization from experimental data [67], such as (i) halogen bond between bromine present in the bowsprit and Y^4i11^; (ii) hydrogen bond between N atom of pyrazole core from starboard side of (*S*)-enantiomer and Q^3o18^. Interestingly, compound **4f** also shows a π-π stacking interaction with F^3i22^, defined as one of the major contributors to the ligand-receptor energy stabilization of 1,4-DHPs at the Ca_V_1.2 l-subtype VDCC model [55]. In summary, the predicted binding mode for compound **4f** is analogous to other 1,4-DHPs such as (*S*)-nimodipine, showing interactions with key DHP-sensing residues of Ca_V_1.2 l-subtype VDCCs.

### 3.4. Neuroprotective Properties of Derivatives ***4a**–**l*** towards Tau-Hyperphosphorylation, [Ca^2+^]_c_ Overload and Oxidative Stress

**Figure 3:**
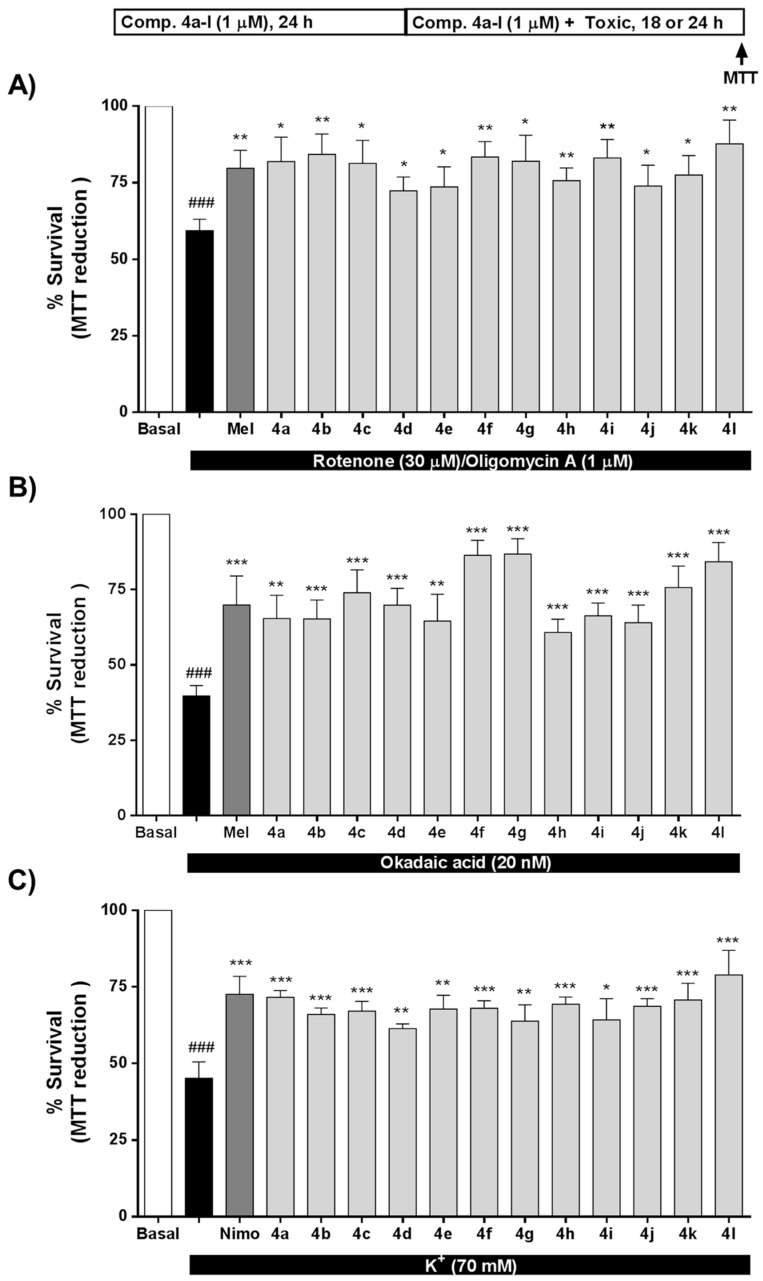
Neuroprotective activity of compounds **4a**–**l** against the toxicity exerted by (**A**) oxidative stress, (**B**) tau hyperphosphorylation and (**C**) [Ca^2+^]_c_ overload. SH-SY5Y neuroblastoma cells were treated with compounds 4a-l (1 μM) or reference compounds (melatonin or nimodipine, 1 μM) during 24 h. Thereafter, cells were co-incubated with compounds 4a-l, melatonin or nimodipine (1 μM) and the corresponding toxic stimuli, R/O combination (30/10 μM, oxidative stress, 24 h), okadaic acid (OA) (20 nM, tau hyperphosphorylation, 18 h) or 70 mM K^+^ ([Ca^2+^]_c_ overload, 24 h). Cell viability was assessed by the 3-(4,5-dimethylthiazol-2-yl)-2,5-diphenyltetrazolium bromide (MTT) reduction assay. Values are expressed as mean ± S.E.M of five independent experiments in triplicate and results were normalized to basal conditions (100%). ^###^ *p* < 0.001 compared to basal condition; * *p* < 0.05, ** *p* < 0.01, *** *p* < 0.001 compared to toxic stimuli treated cells.

As mentioned earlier, OS strongly contributes to AD onset and development, being a key pathological factor in the neurodegeneration observed in this disease [68]. Compounds **4a**–**l** exert significant antioxidant properties (Table 1); thus, we tested the potential neuroprotection towards OS toxicity induced by rotenone (30 μM) and oligomycin A (10 μM) (R/O). Compounds **4a**–**l** were tested at 1 μM concentration including melatonin as positive control and for comparative purposes. Results are expressed as percentage of survival considering non-treated cells as 100% survival. As shown in Figure 3A, all compounds significantly increased SH-SY5Y cells survival compared to the toxic challenge, eliciting protection values ranging from to 32.0% for compound **4d** (Ar = 3-ClPh) to 71.6% of compound **4l** (Ar = 4-Py). Interestingly, compounds **4b** (Ar = 4-FPh), **4g** (Ar = 4-BrPh) and **4l** (Ar = 4-Py), with protective percentages of 62.7%, 59.7% and 71.6%, respectively, showed a better neuroprotective effect than melatonin (52.5%). In this case, considering that the R/O combination induces high amounts of ROS, we observed an interesting correlation between the neuroprotective ability of compounds **4a**–**l** and their capacity to scavenge ROS. In general, the neuroprotective effect increased with higher scavenger capacity, except in the case of compound **4j**. Compounds with ORAC values lower than 0.9 T.eq. showed the lowest percentages of survival (% survival < 75.7%), while compounds showing higher ORAC values presented percentages of survival higher than 78% in all cases. For example, chlorine derivatives **4c**, **4d** and **4e** (*o*-, *m*- and *p*-ClPh, respectively) showed scavenger capacities of 0.94, 0.63 and 0.66 T.eq., respectively, directly correlated with percentages of survival of 81.3%, 72.3% and 73.6%. In this line, derivative **4b** (Ar = 4-FPh) with ROS scavenger ability of 1.86 showed an improvement on cell viability to 84.3%. The same trend can be extracted for bromophenyl, nitrophenyl and pyridyl derivatives.

As summarized in the introduction, GSK-3β is the main kinase related to the formation of neurotoxic hyperphosphorylated tau, the core component of NFTs in AD. Considering the antioxidant and GSK-3β inhibitory properties of compounds **4a**–**l**, we envisaged their evaluation as potential neuroprotective agents in an in vitro model of tau hyperphosphorylation induced by OA. OA inhibits protein phosphatases 1 and 2A [69], increasing the phosphorylation status of tau [70], being an accepted in vitro model of AD. The toxic cascade activated by OA includes increased levels of OS that also contributes to cellular death [71]. All compounds were tested at 1 μM concentration and melatonin was included as positive control [28] and for comparative purposes (Figure 3B). Successfully, compounds **4a**–**l** showed neuroprotective capacity ranging from 34.6% protection of compound **4h** (Ar = 3-NO_2_Ph) to 77.9% protection of compound **4g** (Ar = 4-BrPh). Compounds **4c** (Ar = 2-ClPh), **4f** (Ar = 2-BrPh), **4g** (Ar = 4-BrPh), **4k** (Ar = 3-Py) and **4l** (Ar = 4-Py) showed neuroprotective percentages over 50%, being the compounds **4f**, **4g** and **4l** more potent than the reference compound, melatonin. In this particular model, the neuroprotective effect appears to be dependent on both, the antioxidant effect and the GSK-3β inhibitory potency.

In this line, compound **4g** with the best neuroprotective profile increased cell viability to 86.7%, and showed good GSK-3β inhibition (IC_50_ = 2.35 μM), although it was less potent than compound **4h**, the most potent GSK-3β inhibitor (IC_50_ = 0.82 μM). However, compound **4h** increased cell viability to only 60.8% and, interestingly, the difference between them was the antioxidant capacity, being compound **4g** (ORAC = 0.96 T.eq) more potent than compound **4h** (ORAC = 0.73 T.eq.). Similarly, compound **4c** exhibited an increased survival percentage (74.0%) compared to compound **4e** (percentage survival = 64.6%), compound **4c** being a slightly more potent GSK-3β inhibitor (**4c** IC_50_ = 1.51 μM compared to **4e** IC_50_ = 1.94) and a better antioxidant (**4c** = 0.94 T.eq. compared to **4e** = 0.66 T.eq). These results notwithstanding, and similarly to results obtained in the OS toxicity evaluation, compound **4j** did not follow the same tendency. Although it was the most potent antioxidant (ORAC = 2.93 T.eq.) and a good GSK-3β inhibitor (IC_50_ = 1.74 μM), it increased cell viability to just 64.0%.

Regarding the second target pursued in compounds **4a**–**l**, VDCC antagonism, we evaluated their potential neuroprotective profile against [Ca^2+^]_c_ overload induced by high K^+^ (70 mM). Nimodipine, a potent VDCC antagonist, was included as positive control at 1 μM. As shown in Figure 3C, compounds **4a**–**l** exhibited an interesting neuroprotective profile with survival percentages ranging from 61.4 % of compound **4d** (Ar = 3-ClPh) to 78.9 % for compound **4l** (Ar = 4-Pyridyl). In this case, neuroprotection capacity was similar for all compounds with percentages of survival in the range of 70%, except in the case of compound **4l**. Compounds **4e** and **4f** showed highly potent VDCC antagonism capacity. This potency correlated with interesting neuroprotection capacity (67.6% and 68.0%, respectively); however, they were moderated neuroprotectants compared to compound **4l**. Regarding their antioxidant properties, in this case, the correlation between their neuroprotective capacity against [Ca^2+^]_c_ overload and their scavenger capacity was not clearly correlated. However, the most potent neuroprotectant in this model, compound **4l**, showed moderate VDCC antagonist capacity and high antioxidant ability (ORAC = 1.1 T.eq.) indicating a potential implication in its neuroprotective mechanism of action. Recently, we described the potential dependence of the neuroprotective effect in this toxicity model with the antioxidant effect [72] following results previously published and demonstrating the relationship between Ca^2+^ entry through VDCC and increased levels of free radicals [42,45,73].

In addition to compounds **4a**–**l** neuroprotective profile, we evaluated their safety profile by testing their neurotoxicity in the SH-SY5Y neuroblastoma cell line and their gliotoxicity in primary mixed glial cultures. Our results indicate that compounds **4a**–**l** are neither neurotoxic nor gliotoxic with LD_50_ values higher than 100 μM (Table SI2, Supporting Information).

### 3.5. Compound **4g** Reduces Cellular Death Induced by Okadaic Acid in Hippocampal Slices by Reducing Oxidative Stress

These encouraging results prompted us to test our compounds in a more complex model of AD. For this, we selected an acute treatment with OA of hippocampal slices. Recently, we have demonstrated the deleterious effect of OA (1 μM) in hippocampal slices after 6 h treatment [74]. It increases ROS production, decreases cellular viability and deregulates the autophagy system; thus, it was described as an ex vivo model of tauopathy mimicking AD pathology. For this purpose, we selected compound **4g** (Ar = 4-BrPh) due to its overall pharmacological profile as a potent antioxidant able to scavenge ROS and RNS free radicals, a potent anti-inflammatory properties at low micromolar range, a GSK-3β inhibitor in the same low micromolar range and a moderate VDCC antagonist. The multitarget profile integrated into compound **4g** afforded potent neuroprotective effect against OS and [Ca^2+^]_c_ overload, and it was the most potent compound against tau hyperphosphorylation. Compound **4g** was tested at 10 μM, a concentration established for control and reference compounds used in these ex vivo models. For these experiments, we included melatonin, known to be highly effective in this model, as a reference and positive control.

**Figure 4:**
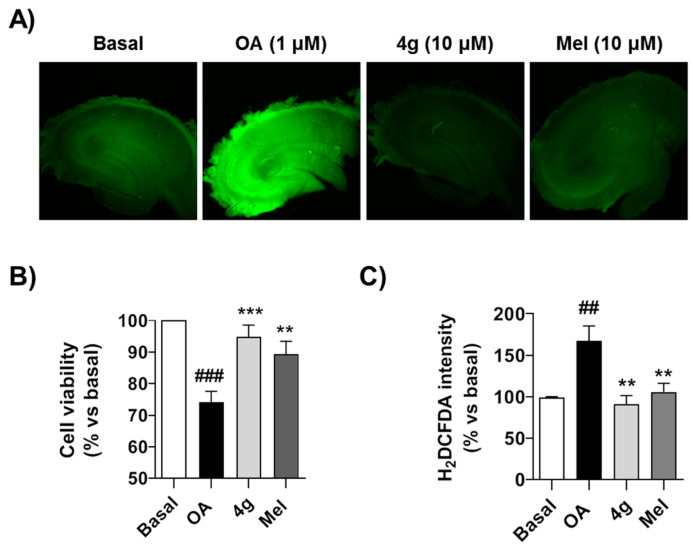
Compound **4g** protects against acute toxicity induced by OA in hippocampal slices: (**A**) Representative H_2_DCFDA fluorescence images of hippocampal slices treated with OA in presence or absence of **4g** (10 μM) or melatonin (10 μM) during 6 h. (**B**) Cell viability of hippocampal slices treated with saline (Basal), OA (1 μM), OA (1 μM) + **4g** (10 μM) or OA (1 μM) + melatonin (**Mel**, 10 μM) during 6 h. Cell viability was then evaluated by MTT reduction assay and normalized to basal conditions (100%). (**C**) Quantitation of H_2_DCFDA fluorescence intensity as free radical production measurement after the experimental conditions described in (**B**). Data are expressed as mean ± S.E.M of four different experiments. ^##^ *p* < 0.01, ^###^ *p* < 0.001 compared to basal condition; ** *p* < 0.01, *** *p* < 0.001, compared to OA treated hippocampal slices.

As shown in Figure 4A, OA reduced cell viability by 30% (*p* < 0.001) measured by the MTT reduction assay. Successfully, compound **4g** recovered cell viability to 92.1%, being a slightly more potent neuroprotector than melatonin, although the differences were not significant (Figure 4B). As demonstrated previously, treatment with OA induces a significant increase in free radical production [74], measured as H_2_DCFDA fluorescence intensity increase (*p* < 0.01), and this ROS increase is related to the toxicity elicited by OA. Under these experimental conditions, compound **4g** effectively reduced free radical production to basal conditions (*p* < 0.01) with an equal potency to melatonin, a well-known antioxidant molecule (Figure 4A,C). As summarized above, we have demonstrated the scavenger capacity of this compound, and thus this antioxidant capacity should be part of its protective mechanism of action.

## 4. Conclusions

AD is currently the most common type of dementia affecting an increasing number of patients and is one of the most important unmet clinical needs for our society. The development of an effective therapy with real disease modifying capacity is an urgent goal for medicinal chemists and biomedical research. The complexity of AD has led to the proposal of the “multitarget hypothesis” for drug development in which a unique chemical entity combines different activities to tackle the pathological pathways involved in AD onset and development. Following this hypothesis, we have obtained a new set of multitarget compounds based on the chemical core of the 1,4-DHPs due to their antioxidant properties and well-known VDCC antagonism capacity. In addition, the fusion of a pyrazole ring generated a suitable scaffold able to inhibit GSK-3β, a kinase related to tau hyperphosphorylation and pro-inflammatory pathways. The combination of pyrazole and 4-aryl-1,4-dihydropyridine moieties into a single molecular entity was achieved through a Hantzsch-like three-component reaction from 3-methyl-1*H*-pyrazol-5-amine, aromatic aldehydes and malononitrile. Compounds **4a**–**l**, thus generated, have demonstrated ROS and RNS antioxidant capacity, anti-inflammatory properties, GSK-3β inhibitory capacity, at low micromolar range and l-type VDCC blockade with micromolar potency. Moreover, the multi-target directed ligands developed were found to lack neurotoxicity or gliotoxicity. They also showed good neuroprotective activity towards OS, tau-hyperphosphorylation and [Ca^2+^]_c_ overload induced toxicity in cell cultures, with interesting correlations between their primary targets and this neuroprotective effect indicating their participation in the neuroprotective mechanism of action. From our set of compounds we have identified 6-amino-4-(4-bromophenyl)-3-methyl-4,7-dihydro-2*H*-pyrazolo[3,4-*b*]pyridine-5-carbonitrile (**4g**) as an interesting multitarget directed ligand for further optimization. Compound **4g** showed good antioxidant activity (ORAC = 0.96 T.eq.; DPPH IC_50_ = 24.1 μM), potent anti-inflammatory capacity (EC_50_ = 4.50 μM), GSK-3β inhibitory capacity (IC_50_ = 2.35 μM) and VDCC antagonist activity (percentage response reduced to 62%) and it is predicted to cross the BBB. Moreover, it showed potent neuroprotective properties against OS, tau hyperphosphorylation and [Ca^2+^]_c_ overload. When tested in a more complex ex vivo model of AD, the acute treatment of hippocampal slices with OA, it demonstrated an extreme reduction of ROS production and also showed potent cytoprotecting capacity. Thus, this compound can be regarded as a good lead for future efforts to increase its potency towards primary targets in a hit to lead project that is currently under development in our laboratories following the promising and novel multitarget approach described here.

## Figures and Tables

**Table 1:** Antioxidant and anti-inflammatory properties of derivatives **4a**–**l** and reference compounds.

Compound	Ar	ORAC (T. Eq.)	IC_50_ DPPH (µM)	EC_50_ Nitrite Reduction (μM)
**Melatonin**	-	2.14 ± 0.12	>5000	-
**Ascorbic ac.**	-	-	61.9 ± 4.96	-
**Sulforaphane**	-	-	-	1.40 ± 0.30 [28]
**4a**	Ph	0.69 ± 0.04	25.2 ± 7.91	5.20 ± 1.89
**4b**	4-FPh	1.86 ± 0.08	22.8 ± 6.68	5.70 ± 0.62
**4c**	2-ClPh	0.94 ± 0.04	23.3 ± 2.60	14.4 ± 2.19
**4d**	3-ClPh	0.63 ± 0.09	22.8 ± 4.63	0.78 ± 1.98
**4e**	4-ClPh	0.66 ± 0.07	20.9 ± 5.91	4.40 ± 0.35
**4f**	2-BrPh	1.00 ± 0.16	21.6 ± 3.99	10.1 ± 0.72
**4g**	4-BrPh	0.96 ± 0.05	24.1 ± 3.74	4.50 ± 0.60
**4h**	3-NO_2_Ph	0.73 ± 0.09	24.7 ± 4.03	3.50 ± 0.29
**4i**	4-NO_2_Ph	0.96 ± 0.05	23.2 ± 4.31	6.10 ± 1.84
**4j**	2-Thienyl	2.93 ± 0.15	20.9 ± 4.23	5.40 ± 0.27
**4k**	3-Pyridyl	1.03 ± 0.16	17.1 ± 2.18	22.3 ± 3.42
**4l**	4-Pyridyl	1.10 ± 0.13	22.9 ± 4.23	15.6 ± 3.35

Free radical scavenger effect was evaluated from dose-response curves of compounds evaluating their ability to reduce reactive oxygen species (ROS) derived free radicals (ORAC) and reactive nitrogen species (RNS) derived free radicals (DPPH). Nitrite production reduction was evaluated by the Griess method in primary glial cultures stimulated with LPS (1 μg/mL). *- not present or not measured.

**Table 2:** GSK-3β inhibition (IC_50_ and Ki values), voltage-dependent Ca^2+^ channels (VDCC) antagonism properties of derivatives **4a**–**l** and reference compounds.

Compound	Ar	IC_50_ GSK3β (µM)	*K_i_* (µM)	VDCC % Response	PAMPA *Pe* (10^−6^ cm s^−1^)	PAMPA Prediction
**SB216763**	-	0.034 ± 0.01 [28]	0.009	-	-	-
**Nimodipine**	-	-	-	51.0 ± 2.6	-	-
**4a**	Ph	18.6 ± 0.4 [28]	13.1	72.1 ± 5.8	8.00 ± 1.5	CNS+
**4b**	4-FPh	2.14 ± 1.1	1.50	48.5 ± 7.6	4.80 ± 2.6	CNS+
**4c**	2-ClPh	1.51 ± 1.1	1.06	58.7 ± 6.8	9.40 ± 3.5	CNS+
**4d**	3-ClPh	1.05 ± 0.3	0.74	76.0 ± 6.5	11.8 ± 3.0	CNS+
**4e**	4-ClPh	1.94 ± 0.5	1.36	6.46 ± 1.6	12.9 ± 3.6	CNS+
**4f**	2-BrPh	0.83 ± 0.3	0.58	7.39 ± 2.0	10.1 ± 5.4	CNS+
**4g**	4-BrPh	2.35 ± 1.1	1.65	62.1 ± 1.6	7.20 ± 2.4	CNS+
**4h**	3-NO_2_Ph	0.82 ± 0.2	0.57	75.5 ± 2.7	2.10 ± 1.0	CNS±
**4i**	4-NO_2_Ph	3.09 ± 0.4	2.17	73.2 ± 2.9	3.10 ± 1.0	CNS±
**4j**	2-Thienyl	1.74 ± 1.0	1.22	60.6 ± 6.8	8.80 ± 4.4	CNS+
**4k**	3-Pyridyl	4.53 ± 2.1	3.18	85.6 ± 5.3	0.70 ± 0.9	CNS-
**4l**	4-Pyridyl	10.2 ± 3.2	7.14	68.9 ± 4.5	5.00 ± 2.9	CNS+

IC_50_ values for GSK-3β inhibition were calculated from concentration-response curves. IC_50_ values were obtained from non-linear regression of percentage of activity vs compound concentration. The apparent equilibrium dissociation constant *K_i_* was calculated from IC_50_ values using the Cheng–Prusoff equation [64,65]. VDCC blockade was evaluated using Fluo-4 Ca^2+^ selective dye in SH-SY5Y cells stimulated with 70 mM K^+^; compounds were tested at 10 μM concentration. Blood–brain barrier (BBB) permeability was evaluated by the Parallel Artificial Membrane Permeation Assay (PAMPA) method.

## Supplementary Materials

The following are available online at https://www.mdpi.com/2076-3921/9/8/650/s1, Table SI1 containing PAMPA model control compounds, Table SI2 showing toxicological evaluation, full methodological description including chemical synthetic protocols, pharmacological experiments and images of the ^1^H and ^13^C NMR spectra images, Figures SI1 and SI2 showing molecular docking experiments of reference compounds.
