# Supplementary material for: Antioxidant, Anti-inflammatory and Neuroprotective Profiles of Novel 1,4-Dihydropyridine Derivatives for the Treatment of Alzheimer’s Disease
Source: Antioxidants (Basel). 2020 Jul 22;9(8):650. doi: 10.3390/antiox9080650 (PMC7463999; doi:10.3390/antiox9080650)
Supplement: Supplementary file 1 [file antioxidants-09-00650-s001.pdf]

Supporting Information

Antioxidant, anti-inflammatory and neuroprotective profiles of novel 1,4-dihydropyridine derivatives for the treatment of Alzheimer's disease.

Patrycja Michalska,[1,3,‡] Paloma Mayo,[1,3,‡] Cristina Fernández-Mendívil,[1,3] Giammarco Tenti,[2] Pablo Duarte,[1,3] Izaskun Buendia,[1,3] María Teresa Ramos,[2] Manuela G. López,[1,3] J. Carlos Menéndez[2] and Rafael León[*1,3]*

[1]Instituto Teófilo Hernando y Departamento de Farmacología y Terapéutica, Facultad de Medicina. Universidad Autónoma de Madrid, 28029 Madrid, Spain.

[2]Unidad de Química Orgánica y Farmacéutica, Departamento de Química en Ciencias Farmacéuticas, Facultad de Farmacia, Universidad Complutense, 28040 Madrid, Spain.

[3]Instituto de Investigación Sanitaria, Servicio de Farmacología Clínica, Hospital Universitario de la Princesa, 28006 Madrid, Spain. E-mail: rafael.leon@uam.es.

‡ These authors contributed equally to this work

TABLE OF CONTENTS

1. Supporting experimental data

1.1. PAMPA control compounds; BBB permeability SI3

1.2. Molecular docking predicted position of **5f** at GSK-3β ATP binding site SI4

1.3. Molecular docking of nimodipine and **5f** at VDCC SI5

1.4. LD_{50} in SH-SY5Y and primary glial culture cells. SI6

2. Experimental protocols SI7

2.1. Synthesis SI7

2.2. Pharmacology SI13

3. Copies of spectra SI22

1.1. PAMPA control compounds; BBB permeability

Table SI1: Prediction of the BBB passive permeability of control compounds expressed as Pe ± SD.

Compound	Pe ± SD (10^{-6} cm s^{-1})	
**Testosterone**	25.7 ± 3.2	CNS (+)
**Corticosterone**	5.4 ± 3.2	CNS (+)
**Verapamil**	19.9 ± 4.2	CNS (+)
**Piroxicam**	3.3 ± 0.1	CNS (+/-)
**Progesterone**	2.2 ± 2.8	CNS (+/-)
**Hydrocortisone**	1.3 ± 0.6	CNS (-)
**Caffeine**	1.1 ± 0.2	CNS (-)
**Ofloxacin**	0.5 ± 0.1	CNS (-)
**Theophylline**	0.26 ± 0.28	CNS (-)

CNS + (high BBB permeability predicted): Pe (x10^{-6} cm s^{-1}) > 4.0

CNS - (low BBB permeability predicted): Pe (x10^{-6} cm s^{-1}) < 2.0

CNS +/- (BBB permeability uncertain): Pe (x10^{-6} cm s^{-1}) between 4.0 to 2

1.2. Molecular docking predicted position of 5f at GSK-3β ATP binding site

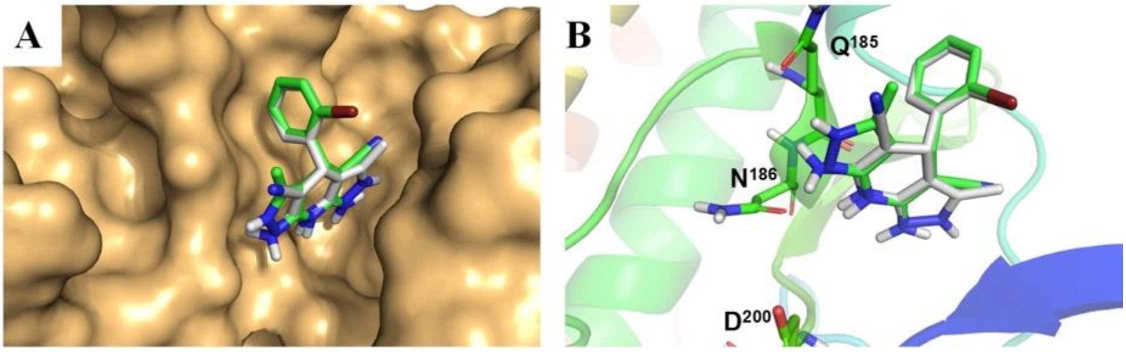

Figure SI1. Molecular docking of compound 4f (*R*)- and (*S*)- enantiomers at the ATP binding site of GSK-3β. (**A**) Overview of compound **4f** docking poses in the ATP binding site. GSK-3β protein represented as orange surface; (**B**) Zoom of merged poses of **4f** (*R*)- and (*S*)- enantiomers represented as white and green sticks, respectively.

1.3. Molecular docking of nimodipine and 4f at VDCC

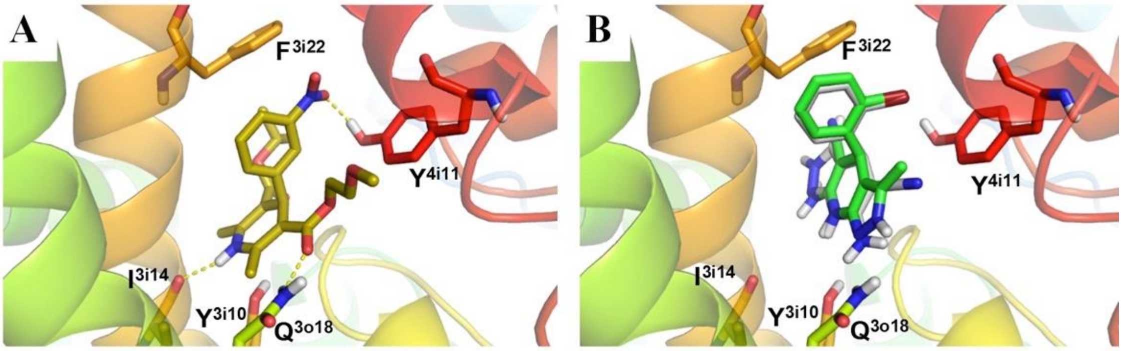

Figure SI2. Molecular docking of compound 4f (*R*)- and (*S*)- enantiomers in the 1,4-DHP binding site of Cav1.2 L-type channel. (**A**) Docked structure of (*S*)-nimodipine represented as gold sticks; **(B)** Merged poses of **4f** *R* and *S* enantiomers represented as white and green sticks, respectively.

1.4. LD_{50} in SH-SY5Y and primary glial culture cells

Table SI2: Cytotoxicity elicited by compounds **4a-l** in the neuroblastoma cell line SH-SY5Y and primary glial cultures. Viability was measured as MTT reduction in presence of increasing concentrations of derivatives. Values are expressed as LD_{50} calculated from dose-response curves of four different concentrations. Data are expressed as mean ± SEM of five different experiments in triplicate.

Compound	R	SH-SY5Y LD_{50} (μM)	Glial cultures LD_{50} (μM)
**Melatonin**	-	>100	>100
**4a**	Ph	>100	>100
**4b**	4-FPh	>100	>100
**4c**	2-ClPh	>100	>100
**4d**	3-ClPh	>100	>100
**4e**	4-ClPh	>100	>100
**4f**	2-BrPh	>100	>100
**4g**	4-BrPh	>100	>100
**4h**	3-NO_2Ph	>100	>100
**4i**	4-NO_2Ph	>100	>100
**4j**	2-thienyl	>100	>100
**4k**	3-Pyridyl	>100	>100
**4l**	4-Pyridyl	>100	>100

2. EXPERIMENTAL PROTOCOLS

2.1. SYNTHESIS

General information

All reagents (Aldrich, Fluka, SDS, Probus) and solvents (SDS) were of commercial quality and were used as received. Reactions were monitored by thin layer chromatography using commercially available aluminum-backed plates coated with silica gel Scharlau Cf 530 with fluorescent indicator and visualized under ultra-violet light lamp Camag UV-II (at 254 and 366 nm). Flash column chromatography was carried out using silica gel SDS 60 ACC or Scharlau Ge 048 and the eluent indicated in each case. Automatic flash chromatography was performed in a Teledyne ISCO COMBI *Flash* Rf instrument using Redi*Sep* Rf silica columns (4 g and 12 g) or self-packed silica cartridges. Melting points were determined using a Stuart Scientific apparatus, SMP3 Model, and are uncorrected. Infrared spectra were recorded on an Agilent Cary630 FTIR spectrometer with a diamond ATR accessory for solid and liquid samples, requiring no sample preparation. NMR spectra were obtained on a Bruker Avance 250 spectrometer operating at 250 MHz for ^{1}H and 63 MHz for ^{13}C (CAI de Resonancia Magnética Nuclear, Universidad Complutense). Gas chromatography mass spectra (GC-MS) were taken with different ionization methods, as Electronic Impact (EI) or Electrospray Ionization (ESI) in both the positive and negative ion mode, and were carried out by SIDI from Universidad Autónoma de Madrid (Madrid, Spain). Quantitative elemental analysis by combustion of carbon, hydrogen, nitrogen and sulfur were carried out in Servicio de Microanálisis Elemental from Universidad Complutense (Madrid, Spain), using a Leco CHNS 932 Elemental Analyzer. The purity of tested compounds was analyzed by CHNS elemental analysis and values were verified to be within 0.4 % of theoretical data.

General procedure for the synthesis of 4,7-dihydro-2*H*-pyrazolo[3,4-*b*]pyridine derivatives (4a-l).

A solution of 3-methyl-1*H*-pyrazol-5-amine **1** (1 equiv, 3 mmol), malononitrile (1 equiv, 3 mmol), arylaldehyde **2** (1 equiv, 3 mmol) and ammonium acetate (1 equiv, 3 mmol) in dry ethanol (5 mL) was heated under reflux. After completion of the reaction (TLC), the solvent was evaporated under reduced pressure and the crude residues were

crystallized from EtOH or purified by flash column chromatography using CH_2Cl_2:MeOH mixtures (100:0 to 90:10 v/v) as eluent to afford pure compounds (**4a-l**).

6-Amino-3-methyl-4-phenyl-4,7-dihydro-2*H*-pyrazolo[3,4-*b*]pyridine-5-carbonitrile (4a).

According to the general procedure, a solution of 3-methyl-1*H*-pyrazol-5-amine **1** (291 mg, 3 mmol), malononitrile (198 mg, 3 mmol), benzaldehyde **2a** (318 mg, 3 mmol) and ammonium acetate (231 mg, 3 mmol) in ethanol (5 mL) was heated under reflux for 16 hours. Thereafter the solvent was evaporated under reduced pressure and the crude residue was crystallized from EtOH to afford compound **4a** as a white solid (0.330 g, 44 % yield). Rf 0.35 (DCM:MeOH 10%); Mp: > 300°C (dec.); IR (neat) ν 3401, 3324, 3225, 2163, 1628 cm^{-1}; ^{1}H NMR (250 MHz, DMSO-d_6) δ 1.75 (s, 3H, C-3CH_3), 4.61 (s, 1H, C*H*-4), 5.44 (s, 2H, C-6NH_2), 7.03-7.21 (m, 3H, C*H*-3', C*H*-4' and C*H*-5'), 7.21-7.39 (m, 2H, C*H*-2' and C*H*-6'), 8.87 (s, 1H, N*H*-7), 11.69 (s, 1H, N*H*-1) ppm; ^{13}C NMR (63 MHz, DMSO-d_6) δ 153.38, 146.87, 146.17, 134.87, 128.25, 127.32, 126.20, 123.30, 100.53, 55.35, 37.79, 9.51 ppm. HRMS (API-ES+) m/z: Cal. for $C_{14}H_{13}N_5$: 251.1171; found: $[(M+H)^+]$ 252.1250; elemental analysis calcd (%) for $C_{14}H_{13}N_5$: C 66.92, H 5.21, N 27.87; found: C 66.83, H 5.21, N 27.69 [1].

6-Amino-4-(4-fluorophenyl)-3-methyl-4,7-dihydro-2*H*-pyrazolo[3,4-*b*]pyridine-5-carbonitrile (4b).

According to the general procedure, a solution of 3-methyl-1*H*-pyrazol-5-amine **1** (291 mg, 3 mmol), malononitrile (198 mg, 3 mmol), 4-fluorobenzaldehyde **2b** (372 mg, 3 mmol) and ammonium acetate (231 mg, 3 mmol) in ethanol (5 mL) was heated under reflux for 16 hours. Thereafter, the solvent was evaporated under reduced pressure and the crude residue was crystallized from EtOH to afford compound **4b** as a white solid (290 mg, 36 % yield). Mp: > 300 °C (dec.); IR (neat) ν 3464, 3191, 3140, 2177, 1599 cm^{-1}; ^{1}H NMR (250 MHz, DMSO-d_6) δ 1.75 (s, 3H, C-3CH_3), 4.65 (s, 1H, CH-4), 5.45 (s, 2H, C-6NH_2), 6.94-7.27 (m, 4H, CH-2', CH-3', CH-5' and CH-6'), 8.89 (s, 1H, NH-7), 11.71 (s, 1H, NH-1) ppm; ^{13}C NMR (63 MHz, DMSO-d_6) δ 160.68 (d, *J* = 241.5 Hz, C-4'), 153.30 (C-6), 146.15 (C-7a), 143.08 (d, *J* = 2.9 Hz, C-1'), 134.85 (C-3), 129.05 (d, *J* = 8.1 Hz, 2xCHAr), 123.17 (CN), 114.93 (d, *J* = 21.2 Hz, 2xCHAr), 100.39 (C-3a), 55.28 (C-5), 36.99 (CH-4), 9.46 (C-3CH_3) ppm; ^{19}F NMR (235 MHz, DMSO-d_6) δ -117.52 (m) ppm; HRMS (API-ES+) m/z: Cal. for $C_{14}H_{12}FN_5$: 269.1077; found: $[(M+H)^+]$ 270.1140;

elemental analysis calcd (%) for $C_{14}H_{12}FN_5$: C 62.44, H 4.49, N 26.01; found: C 62.31, H 4.48, N 25.94.

6-Amino-4-(2-chlorophenyl)-3-methyl-4,7-dihydro-2*H*-pyrazolo[3,4-*b*]pyridine-5-carbonitrile (4c).

According to the general procedure, a solution of 3-methyl-1*H*-pyrazol-5-amine **1** (291 mg, 3 mmol), malononitrile (198 mg, 3 mmol), 2-chlorobenzaldehyde **2c** (422 mg, 3 mmol) and ammonium acetate (231 mg, 3 mmol) in ethanol (5 mL) was heated under reflux for 16 hours. Thereafter, the solvent was evaporated under reduced pressure and the crude residue was crystallized from EtOH to afford compound **4c** as a white solid (290 mg, 34 % yield). Mp: 267-268 °C (dec.); IR (neat) ν 3406, 3313, 3212, 2175, 1636, 1045 cm^{-1}; ^{1}H NMR (250 MHz, DMSO-d_6) δ 1.73 (s, 3H, C-3CH_3), 5.14 (s, 1H, CH-4), 5.48 (s, 2H, C-6NH_2), 7.11-7.32 (m, 3H, CH-4’, CH-5’ and CH-6’), 7.37 (dd, *J* = 7.7, 1.2 Hz, 1H, CH-3’), 8.95 (s, 1H, NH-7), 11.72 (s, 1H, NH-1) ppm. ^{13}C NMR (63 MHz, DMSO-d_6) δ 153.70, 146.38, 143.60, 134.65, 131.19, 130.92, 129.02, 127.96, 127.68, 122.78, 99.92, 54.17, 34.40, 9.27 ppm; HRMS (API-ES+) m/z: Cal. for $C_{14}H_{12}ClN_5$: 285.0781; found: [$(M+H)^+$] 286.0843; elemental analysis calcd (%) for $C_{14}H_{12}ClN_5$: C 58.85, H 4.23, N 24.51; found: C 58.76, H 4.13, N 24.38.

6-Amino-4-(3-chlorophenyl)-3-methyl-4,7-dihydro-2*H*-pyrazolo[3,4-*b*]pyridine-5-carbonitrile (4d).

According to the general procedure, a solution of 3-methyl-1*H*-pyrazol-5-amine **1** (291 mg, 3 mmol), malononitrile (198 mg, 3 mmol), 3-chlorobenzaldehyde **2d** (422 mg, 3 mmol) and ammonium acetate (231 mg, 3 mmol) in ethanol (5 mL) was heated under reflux for 16 hours. Thereafter, the solvent was evaporated under reduced pressure and the crude residue was crystallized from EtOH to afford compound **4d** as a white solid (335 mg, 439 % yield). Mp: 281-282 °C (dec.); IR (neat) ν 3398, 3272, 3194, 2164, 1625, 1074 cm^{-1}; ^{1}H NMR (250 MHz, DMSO-d_6) δ 1.78 (s, 3H, C-3CH_3), 4.67 (s, 1H, CH-4), 5.51 (s, 2H, C-6NH_2), 7.09-7.16 (m, 2H, CH-2’ and CH-6’), 7.21-7.27 (m, 1H, CH-4’), 7.32 (t, *J* = 7.9 Hz, 1H, CH-5’), 8.94 (s, 1H, NH-7), 11.76 (s, 1H, NH-1) ppm; ^{13}C NMR (63 MHz, DMSO-d_6) δ 153.51, 149.48, 146.15, 134.93, 132.90, 130.24, 126.99, 126.26, 126.05, 123.07, 99.92, 54.75, 37.39, 9.46 ppm; HRMS (API-ES+) m/z: Cal. for $C_{14}H_{12}ClN_5$: 285.0781; found: [$(M+H)^+$] 286.0866; elemental analysis calcd (%) for $C_{14}H_{12}ClN_5$: C 58.85, H 4.23, N 24.51; found: C 58.62, H 4.24, N 24.39.

6-Amino-4-(4-chlorophenyl)-3-methyl-4,7-dihydro-2*H*-pyrazolo[3,4-*b*]pyridine-5-carbonitrile (4e).

According to the general procedure, a solution of 3-methyl-1*H*-pyrazol-5-amine **1** (291 mg, 3 mmol), malononitrile (198 mg, 3 mmol), 4-chlorobenzaldehyde **2e** (422 mg, 3 mmol) and ammonium acetate (231 mg, 3 mmol) in ethanol (5 mL) was heated under reflux for 16 hours. Thereafter, the solvent was evaporated under reduced pressure and the crude residue was crystallized from EtOH to afford compound **4e** as a white solid (410 mg, 48 % yield). Mp: 297-298 °C (dec.); IR (neat) ν 3211, 3074, 2167, 1627, 1087 cm^{-1}; ^{1}H NMR (250 MHz, DMSO-d_6) δ 1.76 (s, 3H, C-3CH_3), 4.65 (s, 1H, CH-4), 5.48 (s, 2H, C-6NH_2), 7.15 (d, *J* = 8.4 Hz, 2H, CH-2' and CH-6'), 7.34 (d, *J* = 8.4 Hz, 2H, CH-3' and CH-5'), 8.92 (s, 1H, NH-7), 11.73 (s, 1H, NH-1) ppm; ^{13}C NMR (63 MHz, DMSO-d_6) δ 153.39, 146.15, 145.86, 134.94, 130.66, 129.16, 128.26, 123.12, 100.08, 54.99, 37.14, 9.49 ppm; HRMS (API-ES+) m/z: Cal. for $C_{14}H_{12}ClN_5$: 285.0781; found: $[(M+H)^+]$ 286.0855; elemental analysis calcd (%) for $C_{14}H_{12}ClN_5$: C 58.85, H 4.23, N 24.51; found: C 58.81, H 4.12, N 24.58.

6-Amino-4-(2-bromophenyl)-3-methyl-4,7-dihydro-2*H*-pyrazolo[3,4-*b*]pyridine-5-carbonitrile (4f).

According to the general procedure, a solution of 3-methyl-1H-pyrazol-5-amine **1** (291 mg, 3 mmol), malononitrile (198 mg, 3 mmol), 2-bromobenzaldehyde **2f** (555 mg, 3 mmol) and ammonium acetate (315 mg, 3 mmol) in ethanol (5 mL) was heated under reflux for 16 hours. Thereafter, the solvent was evaporated under reduced pressure and the crude residue was crystallized from EtOH to afford compound **4f** as a white solid (315 g, 32 % yield). Mp: 236-237 °C (dec.); IR (neat) ν 3339, 3216, 3055, 2153, 1626, 1023 cm^{-1}; ^{1}H NMR (250 MHz, DMSO-d_6) δ 1.73 (s, 3H, C-3CH_3), 5.13 (s, 1H, CH-4), 5.48 (s, 2H, C-6NH_2), 6.94-7.17 (m, 2H, CH-5' and CH-6'), 7.32 (t, *J* = 7.0 Hz, 1H, CH-4'), 7.53 (d, *J* = 7.9 Hz, 1H, CH-3'), 8.95 (s, 1H, NH-7), 11.73 (s, 1H, NH-1) ppm; ^{13}C NMR (63 MHz, DMSO-d_6) δ 153.60, 146.31, 145.33, 134.71, 132.14, 131.18, 128.31, 122.67, 121.76, 100.14, 54.47, 36.98, 9.43 ppm; HRMS (API-ES+) m/z: Cal. for $C_{14}H_{12}BrN_5$: 329.0276; found: $[(M+H)^+]$ 330.0365; elemental analysis calcd (%) for $C_{14}H_{12}BrN_5$: C 50.93, H 3.66, N 21.21; found: C 50.77, H 3.66, N 21.08.

6-Amino-4-(4-bromophenyl)-3-methyl-4,7-dihydro-2*H*-pyrazolo[3,4-*b*]pyridine-5-carbonitrile (4g).

According to the general procedure, a solution of 3-methyl-1*H*-pyrazol-5-amine **1** (291 mg, 3 mmol), malononitrile (198 mg, 3 mmol), 4-bomobenzaldehyde **2g** (555 mg, 3 mmol) and ammonium acetate (231 mg, 3 mmol) in ethanol (5 mL) was heated under reflux for 16 hours. Thereafter, the solvent was evaporated under reduced pressure and

the crude residue was crystallized from EtOH to afford compound **4g** as a white solid (505 mg, 51 % yield). Mp: > 300 °C (dec.); IR (neat) ν 3430, 3214, 3070, 2161, 1626, 1069 cm^{-1}; ^{1}H NMR (250 MHz, DMSO-d_6) δ 1.76 (s, 3H, C-3CH_3), 4.63 (s, 1H, CH-4), 5.48 (s, 2H, C-6NH_2), 7.09 (d, *J* = 8.2 Hz, 2H, CH-2' and CH-6'), 7.47 (d, *J* = 8.2 Hz, 2H, CH-3' and CH-5'), 8.91 (s, 1H, NH-7), 11.73 (s, 1H, NH-1) ppm; ^{13}C NMR (63 MHz, DMSO-d_6) δ 153.40, 146.28, 146.16, 134.91, 131.17, 129.56, 123.10, 119.16, 100.02, 54.90, 37.20, 9.47 ppm; HRMS (API-ES+) m/z: Cal. for $C_{14}H_{12}BrN_5$: 329.0276; found: $[(M+H)^+]$ 330.0375; elemental analysis calcd (%) for $C_{14}H_{12}BrN_5$: C 50.93, H 3.66, N 21.21; found: C 50.78, H 3.46, N 21.22.

6-Amino-3-methyl-4-(3-nitrophenyl)-4,7-dihydro-2*H*-pyrazolo[3,4-*b*]pyridine-5-carbonitrile (4h).

According to the general procedure, a solution of 3-methyl-1*H*-pyrazol-5-amine **1** (291 mg, 3 mmol), malononitrile (198 mg, 3 mmol), 3-nitrobenzaldehyde **2h** (453 mg, 3 mmol) and ammonium acetate (231 mg, 3 mmol) in ethanol (5 mL) was heated under reflux for 16 hours. Thereafter, the solvent was evaporated under reduced pressure and the crude residue was crystallized from EtOH to afford compound **4h** as a yellow solid (355 mg, 40 % yield). Mp: 249-250 °C (dec.); IR (neat) ν 3357, 3162, 2161, 1644, 1594, 1340 cm^{-1}; ^{1}H NMR (250 MHz, DMSO-d_6) δ 1.77 (s, 3H, C-3CH_3), 4.87 (s, 1H, CH-4), 5.58 (s, 2H, C-6NH_2), 7.56-7.67 (m, 2H, CH-5' and CH-6'), 7.96 (s, 1H, CH-2'), 8.07 (dt, *J* = 6.7, 2.2 Hz, 1H, CH-4'), 9.02 (s, 1H, NH-7), 11.80 (s, 1H, NH-1) ppm; ^{13}C NMR (63 MHz, DMSO-d_6) δ 153.67, 149.23, 147.89, 146.17, 135.16, 134.17, 130.01, 122.98, 121.58, 121.46, 99.56, 54.44, 37.29, 9.48 ppm; HRMS (API-ES+) m/z: Cal. for $C_{14}H_{12}N_6O_2$: 296.1022; found: $[(M+H)^+]$ 297.1105; elemental analysis calcd (%) for $C_{14}H_{12}N_6O_2$: C 56.75, H 4.08, N 28.36; found: C 56.77, H 4.01, N 28.17.

6-Amino-3-methyl-4-(4-nitrophenyl)-4,7-dihydro-2*H*-pyrazolo[3,4-*b*]pyridine-5-carbonitrile (4i).

According to the general procedure, a solution of 3-methyl-1*H*-pyrazol-5-amine **1** (291 mg, 3 mmol), malononitrile (198 mg, 3 mmol), 4-nitrobenzaldehyde **2i** (453 mg, 3 mmol) and ammonium acetate (231 mg, 3 mmol) in ethanol (5 mL) was heated under reflux for 16 hours. Thereafter, the solvent was evaporated under reduced pressure and the crude residue was crystallized from EtOH to afford compound **4i** as a yellow solid (0.310 mg, 35 % yield). Mp: > 300 °C (dec.); IR (neat) ν 3440, , 3220, 2165, 1625, 1597, 1350 cm^{-1}; ^{1}H NMR (250 MHz, DMSO-d_6) δ 1.77 (s, 3H, C-3CH_3), 4.83 (s, 1H, CH-4),

5.58 (s, 2H, C-6NH$_2$), 7.41 (d, *J* = 7.8 Hz, 2H, CH-2’ and CH-6’), 8.18 (d, *J* = 7.8 Hz, 2H, CH-3’ and CH-5’), 9.02 (s, 1H, NH-7), 11.80 (s, 1H, NH-1) ppm; ^{13}C NMR (63 MHz, DMSO-*d$_6$)* δ 154.42, 153.66, 146.16, 146., 135.14, 128.52, 123.82, 122.92, 99.42, 54.18, 37.52, 9.45 ppm; HRMS (API-ES+) m/z: Cal. for $C_{14}H_{12}N_6O_2$: 296.1022; found: [(M+H)$^+$] 297.1137; elemental analysis calcd. (%) for $C_{14}H_{12}N_6O_2$: C 56.75, H 4.08, N 28.36; found: C 56.74, H 4.01, N 28.17.

6-Amino-3-methyl-4-(thiophen-2-yl)-4,7-dihydro-2*H*-pyrazolo[3,4-*b*]pyridine-5-carbonitrile (4j).

According to the general procedure, a solution of 3-methyl-1*H*-pyrazol-5-amine **1** (291 mg, 3 mmol), malononitrile (198 mg, 3 mmol), 2-thiophenecarboxaldehyde **2j** (336 mg, 3 mmol) and ammonium acetate (231 mg, 3 mmol) in ethanol (5 mL) was heated under reflux for 16 hours. Thereafter, the solvent was evaporated under reduced pressure and the crude residue was crystallized from EtOH to afford compound **4j** as a white solid (185 mg, 24 % yield). Mp: 275 -276 °C (dec.); IR (neat) ν 3390, 3330, 3061, 2164, 1627 cm^{-1}. ^{1}H NMR (250 MHz, DMSO-*d$_6$)* δ 1.89 (s, 3H, C-3CH$_3$), 5.00 (s, 1H, CH-4), 5.48 (s, 2H, C-6NH$_2$), 6.73-7.01 (m, 2H, CH-3’ and CH-4’), 7.31 (dd, *J* = 4.5, 1.7 Hz, 1H, CH-5’), 8.93 (s, 1H, NH-7), 11.76 (s, 1H, NH-1) ppm; ^{13}C NMR (63 MHz, DMSO-*d$_6$)* δ 153.21, 152.56, 145.70, 135.29, 126.30, 124.39, 123.06, 100.34, 55.61, 32.83, 9.50 ppm; HRMS (API-ES+) m/z: Cal. for $C_{12}H_{11}N_5S$: 257.0735; found: [(M+H)$^+$] 258.0834; elemental analysis calcd (%) for $C_{12}H_{11}N_5S$: C 56.01, H 4.31, N 27.22, S 12.46; found: C 56.06, H 4.24, N 27.04, S 12.35.

6-Amino-3-methyl-4-(pyridin-3-yl)-4,7-dihydro-2*H*-pyrazolo[3,4-*b*]pyridine-5-carbonitrile (4k).

According to the general procedure, a solution of 3-methyl-1*H*-pyrazol-5-amine **1** (291 mg, 3 mmol), malononitrile (198 mg, 3 mmol), 3-pyridinecarboxaldehyde **2k** (321 mg, 3 mmol) and ammonium acetate (231 mg, 3 mmol) in ethanol (5 mL) was heated under reflux for 16 hours. Thereafter, the solvent was evaporated under reduced pressure and the crude residue was crystallized from EtOH to afford compound **4k** as a white solid (370 g, 49 % yield). Mp: 290-291 °C (dec.); IR (neat) ν 3389, 3325, 3191, 2158, 1625 cm^{-1}; ^{1}H NMR (250 MHz, DMSO-*d$_6$)* δ 1.75 (s, 3H, C-3CH$_3$), 4.70 (s, 1H, CH-4), 5.52 (s, 2H, C-6NH$_2$), 7.32 (dd, *J* = 7.8, 4.7 Hz, 1H, CH-2’), 7.48 (dt, *J* = 7.8, 1.8 Hz, 1H, CH-6’), 8.40 (dd, *J* = 4.7, 1.8 Hz, 2H, CH-4’ and CH-5’), 8.95 (s, 1H, NH-7), 11.76 (s, 1H, NH-1) ppm; ^{13}C NMR (63 MHz, DMSO-*d$_6$)* δ 153.56, 148.50, 147.70, 146.17, 141.94, 134.97, 123.76, 123.05, 99.65, 54.50, 35.18, 9.44 ppm; HRMS (API-ES+) m/z: Cal. for

$C_{14}H_{12}N_6$: 252.1123; found: [(M+H)$^+$] 253.1215; elemental analysis calcd. (%) for $C_{14}H_{12}N_6$: C 61.89, H 4.79, N 33.31; found: C 61.72, H 4.81, N 32.97.

6-Amino-3-methyl-4-(pyridin-4-yl)-4,7-dihydro-2*H*-pyrazolo[3,4-*b*]pyridine-5-carbonitrile (4l).

According to the general procedure, a solution of 3-methyl-1*H*-pyrazol-5-amine **1** (291 mg, 3 mmol), malononitrile (198 mg, 3 mmol), 4-pyridinecarboxaldehyde **2l** (321 mg, 3 mmol) and ammonium acetate (231 mg, 3 mmol) in ethanol (5 mL) was heated under reflux for 16 hours. Thereafter, the solvent was evaporated under reduced pressure and the crude residue was crystallized from EtOH to afford compound **4l** as a white solid (395 mg, 52 % yield). Mp: > 300 °C (dec.); IR (neat) ν 3456, 3365, 3189, 2166, 1628 cm^{-1}; ^{1}H NMR (250 MHz, DMSO-d_6) δ 1.78 (s, 3H, C-3CH_3), 4.66 (s, 1H, CH-4), 5.55 (s, 2H, C-6NH_2), 7.13 (dd, *J* = 4.5, 1.5 Hz, 2H, CH-3' and CH-5'), 8.47 (dd, *J* = 4.5, 1.5 Hz, 2H, CH-2' and CH-6'), 8.97 (s, 1H, NH-7), 11.78 (s, 1H, NH-1) ppm; ^{13}C NMR (63 MHz, DMSO-d_6) δ 154.93, 153.75, 149.73, 146.16, 135.08, 122.92, 122.57, 99.08, 53.81, 37.10, 9.45 ppm; HRMS (API-ES+) m/z: Cal. for $C_{14}H_{12}N_6$: 252.1123; found: [(M+H)$^+$] 253.1212; elemental analysis calcd (%) for $C_{14}H_{12}N_6$: C 61.89, H 4.79, N 33.31; found: C 61.83, H 4.71, N 33.10.

2.2. PHARMACOLOGY

Minimal essential medium (MEM), EMEM, DMEM with GlutaMAX, fetal bovine serum (FBS), geneticin, and Hank's balanced salt solution were acquired from Life Technologies (Madrid, Spain). Rotenone, oligomycin A, okadaic acid, penicillin/streptomycin, sodium pyruvate, NEDA (N-(1-Naphthyl)ethylenediamine dihydrochloride), DAPSONE (4,4´-diamino-di-phenylsulfone), lipopolysaccharide (LPS), 2,2'-azobis(amidinopropane) dihydrochloride, (3-(4,5-dimethylthizaol-2-yl)-2,5-diphenyltetrazoliumbromide (MTT), ascorbic acid, melatonin, Trolox ((±)-6-hydroxy-2,5,7,8-tetramethylchromane-2-carboxylic acid) and SB216763 were purchased from Sigma-Aldrich (Madrid, Spain). GSK-3β Kinase Enzyme System (V1991), Luciferase Assay System (E1500) and Kinase-Glo Luminescent Kinase Assay (V6712) were purchased from Promega (Madison, WI, USA). GSK-3β peptide substrate was purchased from Millipore (Millipore Iberica, Madrid, Spain).

Ethical issues on the use of animals

All experimental procedures were performed following the *Guide for the Care and Use of Laboratory Animals* and were previously approved by the institutional Ethics Committee of the Autonomous University of Madrid, Spain, according to the European Guidelines for the use and care of animals for research in accordance with the European Union Directive of 22 September 2010 (2010/63/UE) and with Spanish Royal Decree of 1 February 2013 (53/2013). All efforts were made to minimize animal suffering and to reduce the number of animals used. Animals were housed under controlled conditions (22 ± 1ºC, 55-65 % humidity, 12 h light dark cycle), with free access to water and standard laboratory chow. We have used Sprague-Dawley rats (2-3 days postnatal) supplied by the animal facilities of Universidad Autónoma de Madrid (Madrid, Spain).

Oxygen Radical Absorbance Capacity (ORAC) assay

ORAC test developed by Cao *et al.* [2] and modified by Ou *et al.* [3] was carried out to evaluate the oxygen free radical scavenger capacity of the compounds. (±)-6-hydroxy-2,5,7,8-tetramethylchromane-2-carboxylic acid (Trolox) (1, 2, 4, 6 and 8 µM) was used as reference compound and melatonin as positive control. Compounds **4a-l** were diluted (0.03, 0.1, 0.3, 1, 3 and 10 µM) in PBS buffer (10 mM, pH 7.4) at 37 ˚C and placed a 96-well black microplate (COSTAR 3904). 150 µL of fluorescein (70 nM, final concentration), 25 µL of PBS buffer for blank, 25 µL of Trolox solution for standard and 25 µL of melatonin or compound were added to each well. A fluorescence measurement

was done first to determine the basal signal. Then, 25 µL of 2,2´-azobis-amidinopropane dihydrochloride (AAPH) (12 mM, final concentration) were quickly added, since the reaction starts immediately after addition. All samples were carried out in duplicate in three different experiments. Fluorescence was recorded every min for 90 min at 37 ˚C to obtain the area under the fluorescence decay curve (fluorescence vs time) (AUC) in a FLUOstar Optima plate reader (BMG Labtech, Offenburg, Germany) with 485 nm excitation and 520 nm emission filters. After blank correction and plotting the net AUC vs antioxidant concentration, linear regressions were calculated for all the samples. Final results were expressed in Trolox equivalents (T.eq.), where the ORAC value of Trolox was taken as 1.0.

DPPH Reduction Assay

Experimental conditions were modified from a previously described procedure.[4] Briefly, the compounds under assay (150 µL) at the desired concentration (1, 10, 60 and 100 µM) in methanol/water (80/20) were added to a solution of DPPH in methanol/water (80/20) (150 µL, 100 µM), in a clear bottom 96-well plate, and the final solution was incubated 1 h in the dark. Then, DPPH absorbance of blank (methanol/water), control (DPPH 100 µM) and compounds plus DPPH were measured at 540 nm in a FLUOstar Optima plate-reader (BMG Labtech, Ortenberg, Germany) in duplicate. Ascorbic acid, melatonin and Trolox were used as positive controls. Results are expressed as % of absorbance (Abs) reduction of control after subtracting blank absorbance, as expressed below:

$$\% \text{ DPPH reduction} = [100 - (Abs_{sample} - Abs_{blank}) \times 100]/Abs_{control}$$

Mixed glial culture

Mixed glial cultures were obtained from cerebral cortex of 2-5-day-old Sprague-Dawley rats. After removal of blood vessels and meninges, the forebrains were dissociated in DMEM/F12 medium. After mechanical dissociation, cells were plated ($3x10^5$ cells/ml) in DMEM/F12 medium with 20 % fetal bovine serum (FBS) and 1 % penicillin/streptomycin (10,000 units), at 37 ºC and in a 5 % CO_2-supplemented air atmosphere. After 5 days, medium was substituted by DMEM/F12 medium with 10 % FBS. Cells were cultured for 7-10 days before treatment.

Nitrite production measurement in culture medium of mixed glial cells

Cells were pre-incubated with increasing concentrations (0.1, 1, 3, 10, 30 and 60 µM) of compounds for 24 hours. Then, treatments were removed and cells were incubated

with LPS (1 μg/mL) in presence of each compound at the desired concentration. Each plate included non-treated cells as basal nitrite production, sulforaphane (SFN, 10 μM) was included as positive control. Nitrite production was assessed 18 hours later by modified Griess assay. Briefly, samples (150 µl) were mixed with DAPSONE (75 µl) and NEDA (75 µl), and the mixture was incubated at room temperature for 5 min. Light absorption was measured at 550 nm in a microplate reader (Labtech, Offenburg, Germany). All data were normalized to basal nitrite production, considering this value as 100 % of nitrite production. EC_{50} values were calculated from dose-response curves represented as percentage of nitrite production reduction induced by the different concentrations of each compound. Concentration response curves were fitted by non-linear regression analysis of individual concentration-response curves using GraphPad Prism software (San Diego, CA, USA). EC_{50} values are expressed as mean ± SEM of four different experiments in triplicate.

GSK-3β inhibition

The method of Baki et al. [5], with some modifications, was followed to analyze the ability of the compounds to inhibit GSK-3β. The tested compounds were dissolved in assay buffer, containing 40 mM Tris (pH 7.5), 20 mM $MgCl_2$, 0.1 mg/ml BSA (bovine serum albumin) and 50 µM dithiothreitol (DTT), to achieve final reaction concentrations of 0.1, 1, 10 and 30 µM. Compound SB216763 was used as reference compound, at a final concentration of 50 nM. The GSK-3β inhibition assay was performed in white 96-well plates, in duplicates. Firstly, 10 µl of enzyme (10 ng) were added to each well, followed by 10 µl of the compounds at the desired concentration. After 30 minutes, 20 µl of a mixture of ATP and GSK-3β-peptide substrate were added to each well, achieving a final concentration of 1 µM of ATP and 25 of µM peptide substrate. After a 60 min incubation at 30 ºC, the enzyme reaction was stopped, and the remaining ATP concentration was measured by adding 40 µl/well of Kinase-Glo reagent. The luminescence was measured after 30 min in an Orion II microplate luminometer (Berthold, Germany) as relative light units (RLU). The luminescence recorded is proportional to the amount of ATP present in each well. GSK-3β activity is proportional to the difference between the total ATP and the remaining ATP after the enzymatic reaction, considering 100 % of activity the corresponding to that difference in absence of inhibitor. IC_{50} values were calculated by non-linear regression analysis of individual concentration-response curves using GraphPad Prism software (San Diego, CA, USA).

GSK-3β kinetic study: Km calculation

Apparent K_i for each compound were calculated from GSK-3β K_m value for ATP, applying the Cheng-Prusoff equation[6,7] in its simplified form: $K_i = IC_{50}/(1+([S]/K_m))$, in which S refers to the ATP concentration and K_m is the Michaelis constant of the substrate for GSK-3β. GSK-3β $K_{m\text{-}ATP}$ under our experimental conditions were calculated using a modified Baki method and a Lineweaver-Burk plot. GSK-3β, ATP, and GSK-3β peptide substrate were dissolved in assay buffer, containing 40 mM Tris (pH 7.5), 20 mM $MgCl_2$, 0.1 mg/ml BSA (bovine serum albumin) and 50 µM dithiothreitol (DTT). The assay was performed in white 96-well plates. To 10 µl of assay buffer, 10 µl (10 ng) of GSK-3β (t = 30 min) or 10 µl of assay buffer (t = 0 min) were added, followed by 20 µl of a mixture of ATP and GSK-3β peptide substrate, achieving a final peptide substrate concentration of 25 µM, and a final ATP concentration of 0, 0.1, 0.4, 1, 4 and 10 µM. The plate was incubated for 30 min at 30 °C. After this time, ATP concentration in absence (t = 0 min) and presence of GSK-3β (t = 30 min) was determined by luminescence after the addition of 40 µl per well of Kinase-Glo reagent. The luminescence was measured after 60 min in an Orion II microplate luminometer (Berthold, Germany) as relative light units (RLU). The luminescence detected is proportional to the ATP concentration in each well. The difference between the total ATP (t = 0 min, absence of enzyme) and the remaining ATP after the enzymatic reaction (t = 30 min) divided for the reaction time (30 min) was considered as the reaction rate (V) for each ATP concentration. To determine the $K_{m\text{-}ATP}$ value, V and ATP concentration values were fitted to a Lineweaver-Burk plot (double reciprocal plot), using Origin software. Ki values were calculated using the Chen-Prusoff equation simplified: $K_i = IC_{50}/(1+(S/K_m))$, being S the substrate concentration (ATP) in the experiment, and K_m the Michaelis constant of the substrate for the enzyme (K_m = 2.345 µM for ATP).

SH-SY5Y neuroblastoma cells culture

SH-SY5Y cells (obtained from ATCC, CRL-2266) were cultured according to supplier directions in a 1:1 mixture of F12 (Ham 12) and Eagle's MEM, supplemented with 15 % non-essential amino acids, 10 % heat-inactivated FBS, 0.5 mM sodium pyruvate, 24 mM $NaHCO_3$, 100 µg/mL streptomycin and 100 units/mL penicillin. Cells were maintained at 37 ºC in humidified atmosphere of 95 % air and 5 % CO_2. For experimental procedures, cells were cultured in 96-well plates ($6x10^5$ cells per well). Treatments were carried out in 1 % FBS medium unless other concentration was specified. Cells were used from 4 up to 13 passages.

Voltage dependent Calcium Channel Blockade Assay

Free cytosolic Ca^{2+} was measured using the fluorescence Ca^{2+} indicator Fluo-4/AM. SH-SY5Y cells were seeded in 96-well black bottom transparent plates at a density of 60,000 cells per well until reaching confluence. Cells were incubated with the Ca^{2+} sensitive dye fluo-4 AM at 5 µM in Krebs-Hepes buffer (KH) (145 mM NaCl, 4.9 mM KCl, 1.2 mM $MgCl_2$, 2 mM $CaCl_2$, 10 mM HEPES, 11 mM glucose, pH 7.4), and 0.05 % pluronic acid, for 1 h at 37 ºC in the dark. Then, cells were washed twice with KH solution to remove the excess of probe and compounds in solution were added. Tested compounds at 10 µM were incubated 10 min before K^+ 70 mM was applied to evoke the increment of cytosolic Ca^{2+}. The experiments were analyzed at excitation and emission wavelengths of 485 and 520 nm, respectively, and fluorescence was measured in a fluorescence microplate reader (FLUOstar Optima, BMG, Germany). To normalize Fluo-4 signals, Triton X-100 (5 %) and subsequently $MnCl_2$ 1 M were applied to cells for registering both maximal and minimal fluorescence, respectively. Data were calculated as % F increment respect to $F_{max} – F_{min}$. % of blockade was calculated considering basal conditions as 100 % response.

Blood-Brain Barrier Permeation Assay

Prediction of the capacity of compounds to cross the blood-brain barrier (BBB) by passive diffusion was evaluated using a Parallel Artificial Membrane Permeation Assay (PAMPA), in a similar manner as described previously [8]. The effective permeability of the compounds was measured in duplicate at an initial concentration of 100 µM. The compounds of interest were dissolved in 10 mM phosphate-buffered saline (PBS) buffer (pH 7.4) to the desired concentration in the donor well (Multiscreen IP sterile clear plate PDVF membrane, pore size 0.45 µM, catalogue no. MAIPS4510, Millipore Corp.). The acceptor 96-well plate (Multiscreen, catalogue no. MAMCS9610, Millipore Corp.) was filled with 180 µL of PBS (V_A). The filter membrane of the donor 96-well plate was impregnated with 4 µL of porcine brain lipid (PBL) (Avanti Polar Lipids, Inc) in dodecane (20 mg/mL) (Sigma-Aldrich, Madrid, Spain), and after 5 min, 180 µL of each compound solution was added to determine their ability to pass the brain barrier (V_D). Then, the donor filter plate was carefully put on the acceptor plate to form a “sandwich”, which was left undisturbed for 4 h at 25 ºC. During this time, the compounds diffused from the donor plate through the polar brain lipid membrane (area = 0.28 cm^2) into the acceptor plate. After incubation, the donor plate was carefully removed. UV plate reader determined the concentration of compounds and commercial drugs in both acceptor and

the donor wells (150 µL/well) as the maximum absorption wavelength of each compound. There were also prepared solutions at the theoretical equilibrium of given compound (i.e. theoretical concentration if the donor and acceptor compartment were simply combined). Concentration of the compounds in the donor and acceptor well and equilibrium concentration were calculated from the standard curve and expressed as the permeability (P_e) according the equation [1]. Results are expressed as mean ± SEM of three different experiments in duplicate. Nine quality control compounds (theophylline, ofloxacin, caffeine, hydrocortisone, piroxicam, corticosterone, progesterone, verapamil and testosterone (Sigma-Aldrich, Madrid, Spain)) of known BBB permeability were included in each experiment to validate the analysis set.

$$\log Pe = \log\left\{C \times -\ln\left(1 - \frac{[drug]_{acceptor}}{[drug]_{equilibrium}}\right)\right\} \quad [1]$$

$$\text{where } C = \left(\frac{V_D \times V_A}{(V_D + V_A) \times Area \times Time}\right)$$

Docking calculations

The GSK-3β structure PDB-ID 3I4B was used to perform molecular docking. Prior to docking calculation, ligand 3D conformations and ligand states at pH 7.2 were obtained with Open Babel software and then minimized using steepest descent method with 1500 steps. Only majority tautomers predicted with ChemAxom platform were considered for calculations. GSK-3β structure was prepared and minimized using MacroModel with Optimized Potentials for Liquid Simulations 3 (OPLS3) force field. After that, molecular docking was performed using AutoDock Vina[9] with center of the box located on ATP-binding site. Best 9 poses were visually inspected with PyMOL (Schrödinger software) and compared by energy. To test our system, we first performed docking of the *N*-[(1*S*)-2-hydroxy-1-phenylethyl]-4-[5-methyl-2-(phenylamino)pyrimidin-4-yl]-1*H*-pyrrole-2-carboxamide inhibitor crystallized in complex with 3i4b structure, obtaining overlapping results with crystal inhibitor position and validating our docking method. The selected 4,7-dihydro-2*H*-pyrazolo[3,4-*b*]pyridine **5f** *R* and *S* enantiomers were submitted to molecular docking with the previous specifications and previous compound **5m** by Gameiro *et* al.[1] was used for comparative purposes. Same docking strategy was used for predictions with $Ca_V1.2$ L-type channel and selected 4,7-dihydro-2*H*-pyrazolo[3,4-*b*]pyridine compound **5f** in the 1,4-Dihydropyridine (DHP) binding site. As crystal structure of this subtype is not available, we used the $Ca_{V1.2}$ L-subtype VDCC

model developed by D. Tikhonov and B. S. Zhorov and kindly shared with us by Prof. Zhorov [10].

Neuroprotection in the SH-SY5Y neuroblastoma cell line

A pre- and co-incubation protocol was followed. Cells were pre-incubated with the corresponding compound at 1 μM in neuroblastoma culture medium. After 24 h, the medium was removed and replaced with 1% FBS neuroblastoma culture media containing the corresponding compound at 1 μM and the toxic stimuli, namely a mixture of rotenone and oligomycin A (30 μM/10 μM respectively), okadaic acid at 20 nM or a high concentration of potassium chloride (70 mM). Cells were co-incubated for further 24 h with the rotenone and oligomycin A or potassium chloride solution, or 18 h with the okadaic acid solution. Control cells were incubated with the same amount of DMSO without any drug. Melatonin (1 μM) or Nimodipine (1 μM) were used as positive control and reference compounds in the rotenone-oligomycin A, okadaic acid or high concentration of potassium models respectively. After the co-incubation period, cell viability was assessed by the MTT-reduction method [11] considering basal conditions as 100 % survival.

Toxicology assay

SH-SY5Y or primary glial cells were cultured in 96-well plates for 24 h. Then, cells were treated with the compounds at 10, 30 and 100 µM in the corresponding culture media with 1 % FBS After 24 h, cell viability was assessed by the MTT-reduction assay. Control cells were incubated with the same concentration of DMSO (0.1 % final concentration) without any drug.

Viability assay by MTT-reduction

SH-SY5Y, primary glial culture cells and rat hippocampal slices viability was measured by the MTT assay [11]. This method is based on the cleavage of the tetrazolium ring of MTT by active dehydrogenases, generating a formazan precipitate. At the end of the neuroprotection or toxicity experiments, an MTT solution was added to each well to reach a final concentration of 0.5 mg/mL. After 2 additional hours of incubation, the solution was replaced by 100 or 300 µL of DMSO to solubilize the formazan precipitate. Absorbance was measured in a NanoStar plate reader at 570 nm. The absorbance obtained in control cells was considered as 100 % of cell survival and used to reference all treatments. The percentage of protection was calculated as follows: the percentage of cell survival obtained with the toxic stimuli-treated cells subtracted to 100 was normalized to

100 % of cell death. The percentage of protection was obtained by subtracting the percentage of cell death in presence of the tested compound to 100 %.

Acute treatment in rat hippocampal slices

3-4 months-old Sprague-Dawley rats were beheaded and both hippocampi dissected and placed in a previously oxygenated (95 % O_2 and 5 % CO_2) ice-cold Krebs´s dissection buffer (120 mM NaCl; 2 mM KCl; 26 mM $NaHCO_3$; 1.18 mM KH_2PO_4; 10 mM $MgSO_4$; 0.5 mM $CaCl_2$; 11 mM glucose and 200 mM sucrose at pH 7.4). Hippocampi were cut into 250 µm thick slices using a McIlwain Tissue Chopper (Cavey Laboratory Engineering, Surrey, United Kingdom) and separated using a Leica SE6 microscope (Leica, Spain). To allow slice recovering from the previous slicing trauma, they were transferred to a vial containing dissection buffer without sucrose, bubbled with 95 % O_2 and 5 % CO_2 for 45 minutes at 34ºC (stabilization). Afterwards, slices were placed in a 48-well plate containing new medium composed of 1:1 control buffer (120 mM NaCl; 2 mM KCl; 26 mM $NaHCO_3$; 1.18 mM KH_2PO_4; 10 mM $MgSO_4$; 2 mM $CaCl_2$ and 11 mM glucose) and DMEM/F12 medium (Invitrogen, Madrid, Spain).

Measurement of ROS production in hippocampal slices

ROS quantification was performed using the fluorescent probe 2´,7´-dichlorodihydrofluorescein diacetate (H_2DCFDA) (Thermo Fisher, Massachusetts, EEUU). Slices were incubated with the probe for 45 minutes at a concentration of 10 µL/mL, in presence of 1 µL/mL of Hoechst at 37 ºC before finalizing the experimental condition used. H_2DCFDA crosses the cell membrane and is hydrolyzed by intracellular esterase to the non-fluorescent form, dichlorodihydrofluorescein. This reacts with ROS to form dichlorofluorescein, a green fluorescent dye. Fluorescence from the cornu Ammonis 1 (CA_1) was measured at an excitation wavelength of 480 nm and an emission of 520 nm for DCFDA, and 350 nm and 460 nm for Hoechst, in an inverted Nikon Eclipse T2000-U microscope (Nikon, Tokyo, Japan), with a 2X objective. NIS-Elements BR 4.10.04 64-bit software was used to analyze the images.

Statistical analysis

All values are expressed as mean ± S.E.M. IC_{50} and LD_{50} values were calculated by non-linear regression analysis of individual concentration-response curves using GraphPad Prism software (San Diego, CA, USA). Analysis of the results was performed by comparison between experimental and control data using one-way ANOVA followed by Newman-Keuls *post-hoc* test when three groups are implicated. Differences were

considered to be statistically significant when $p \leq 0.05$. “n” represents the number of different cultures used or enzyme inhibition assays performed.

3. COPIES OF SPECTRA

6-Amino-4-(4-fluorophenyl)-3-methyl-4,7-dihydro-2*H*-pyrazolo[3,4-*b*]pyridine-5-carbonitrile 4b.

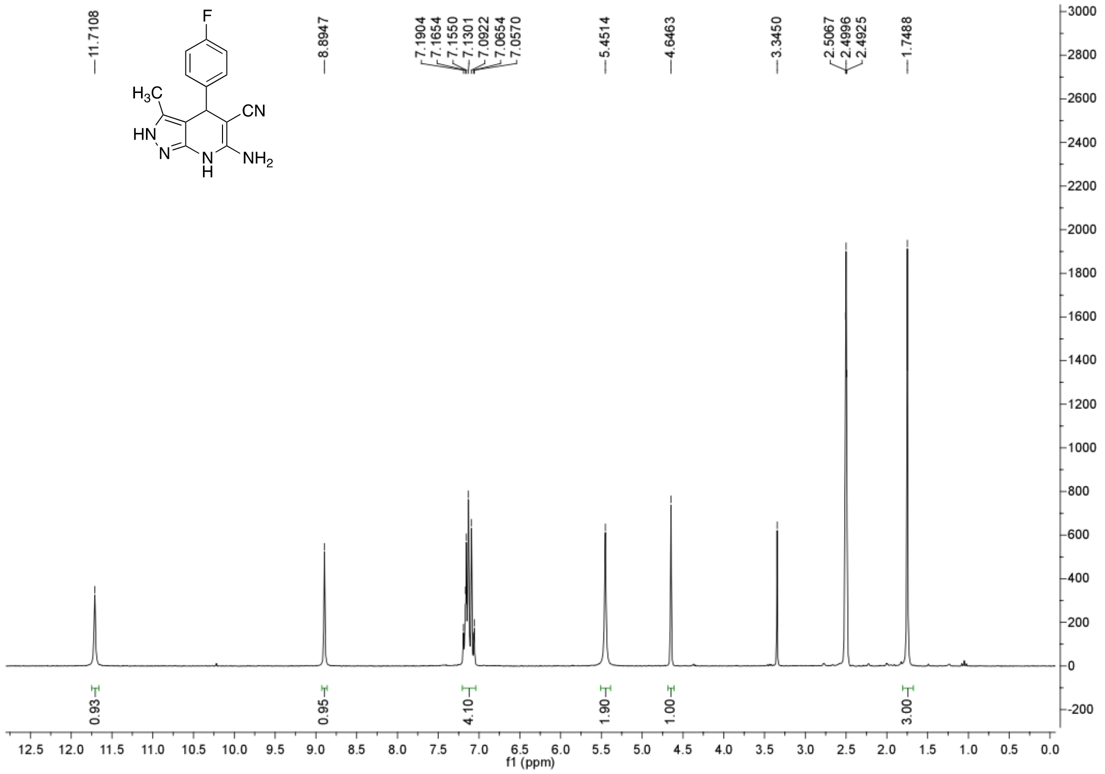

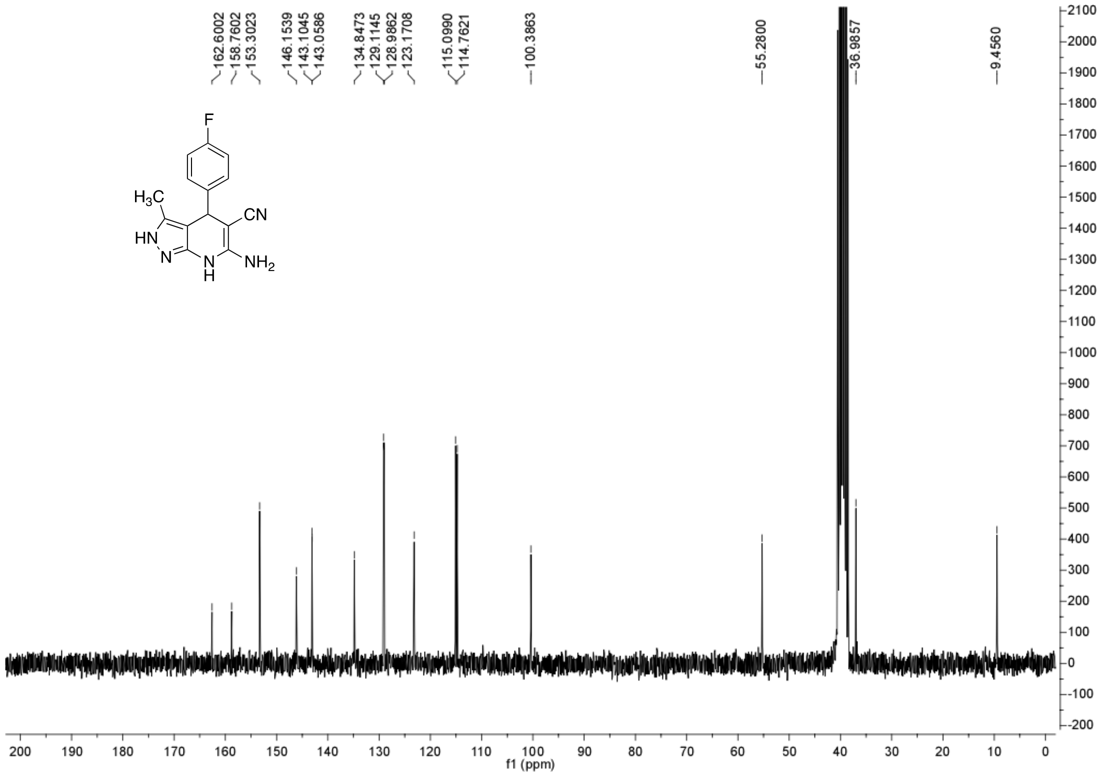

6-Amino-4-(2-chlorophenyl)-3-methyl-4,7-dihydro-2*H*-pyrazolo[3,4-*b*]pyridine-5-carbonitrile 4c.

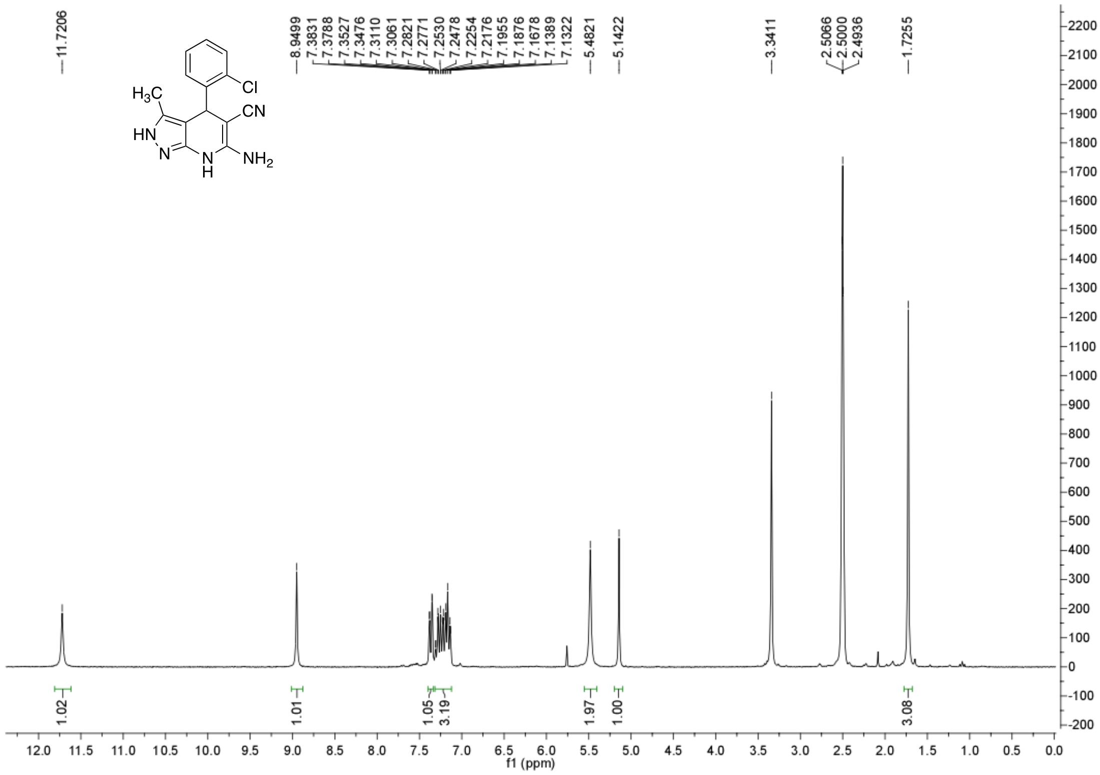

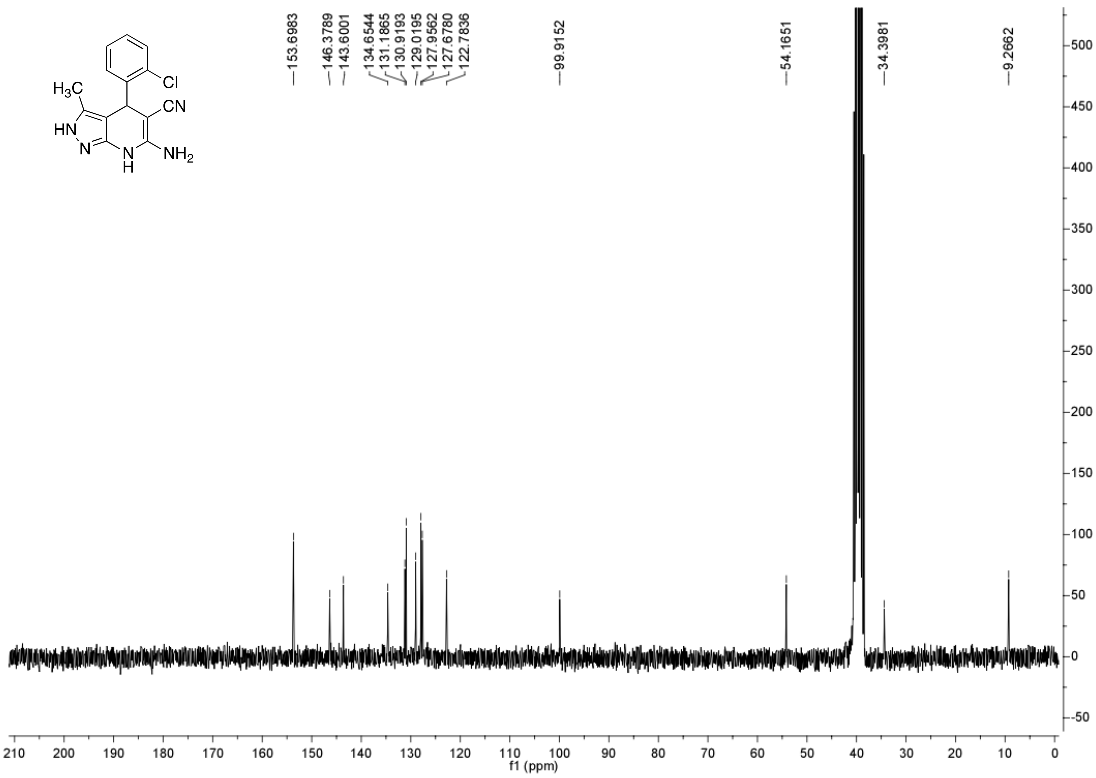

6-Amino-4-(3-chlorophenyl)-3-methyl-4,7-dihydro-2*H*-pyrazolo[3,4-*b*]pyridine-5-carbonitrile 4d.

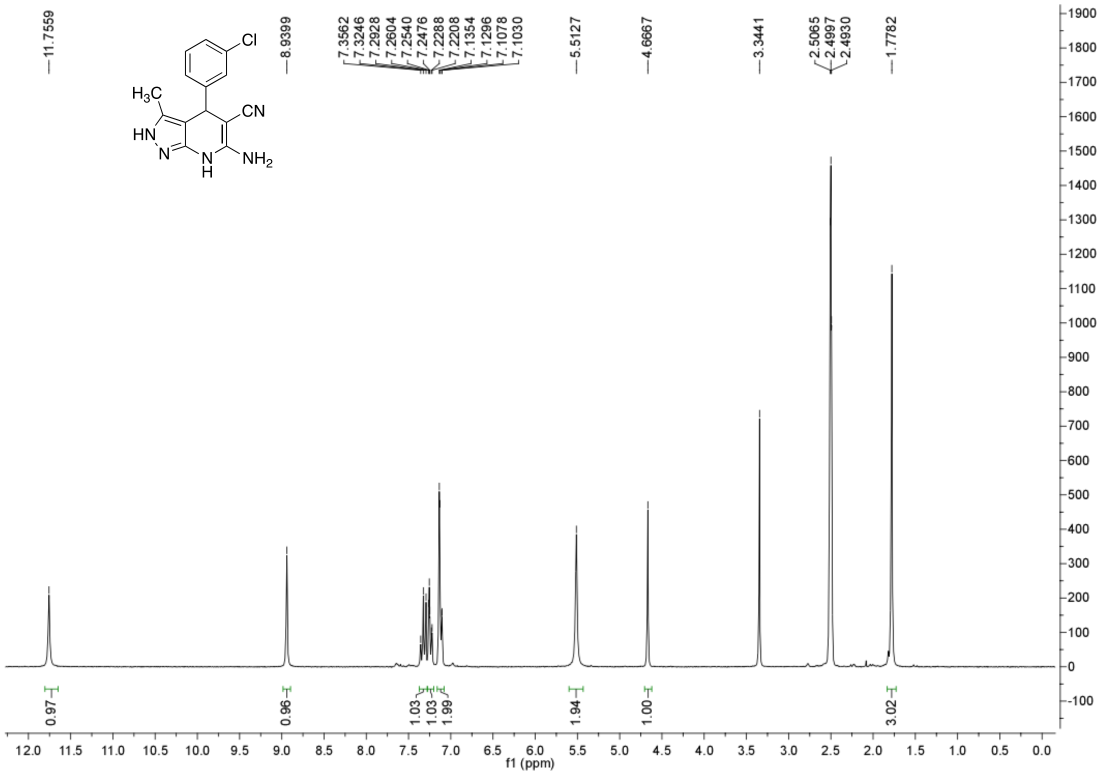

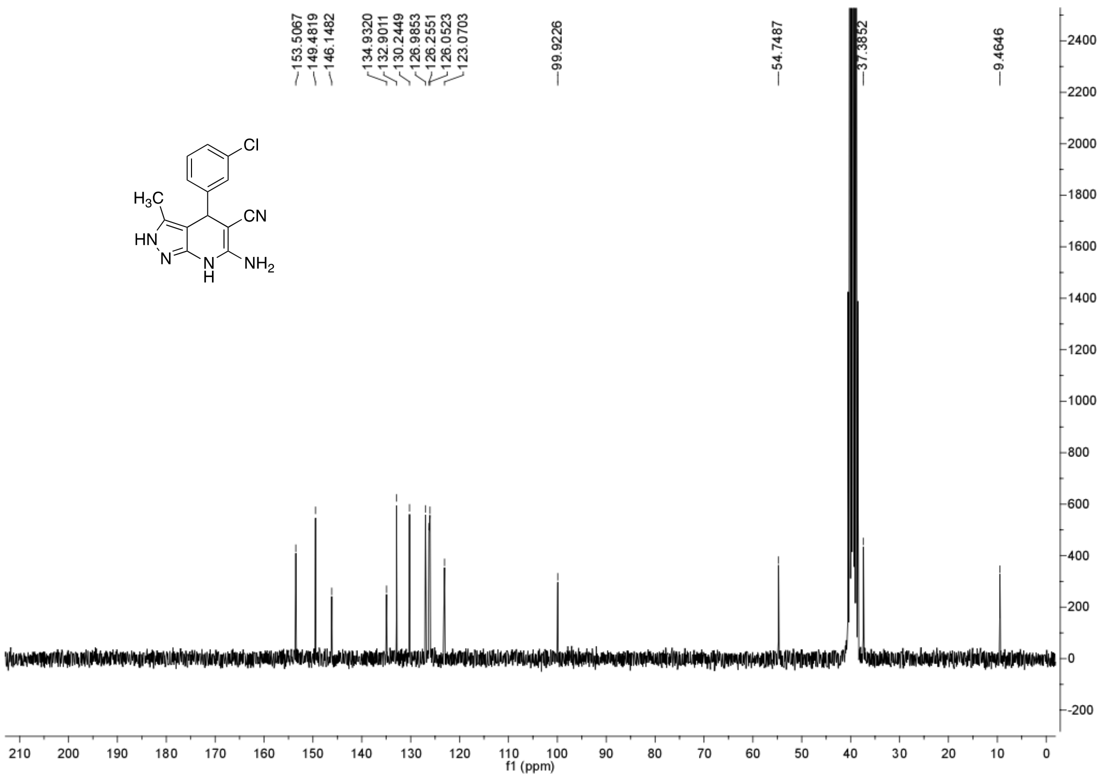

6-Amino-4-(4-chlorophenyl)-3-methyl-4,7-dihydro-2*H*-pyrazolo[3,4-*b*]pyridine-5-carbonitrile 4e.

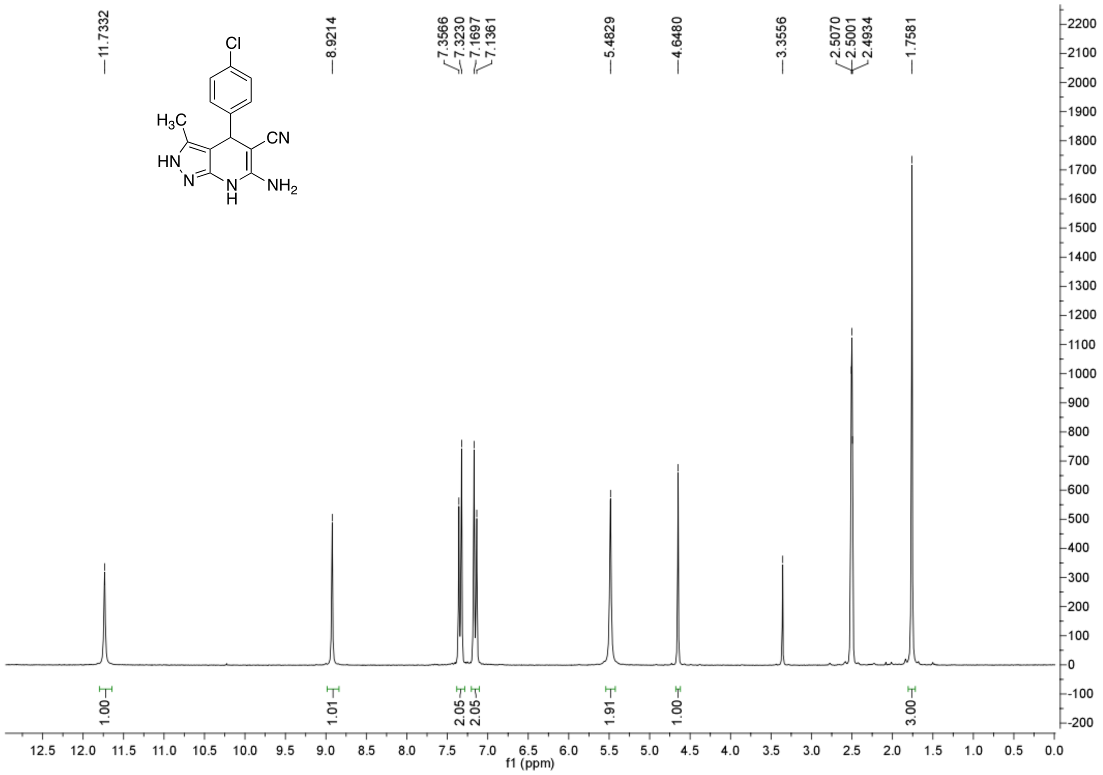

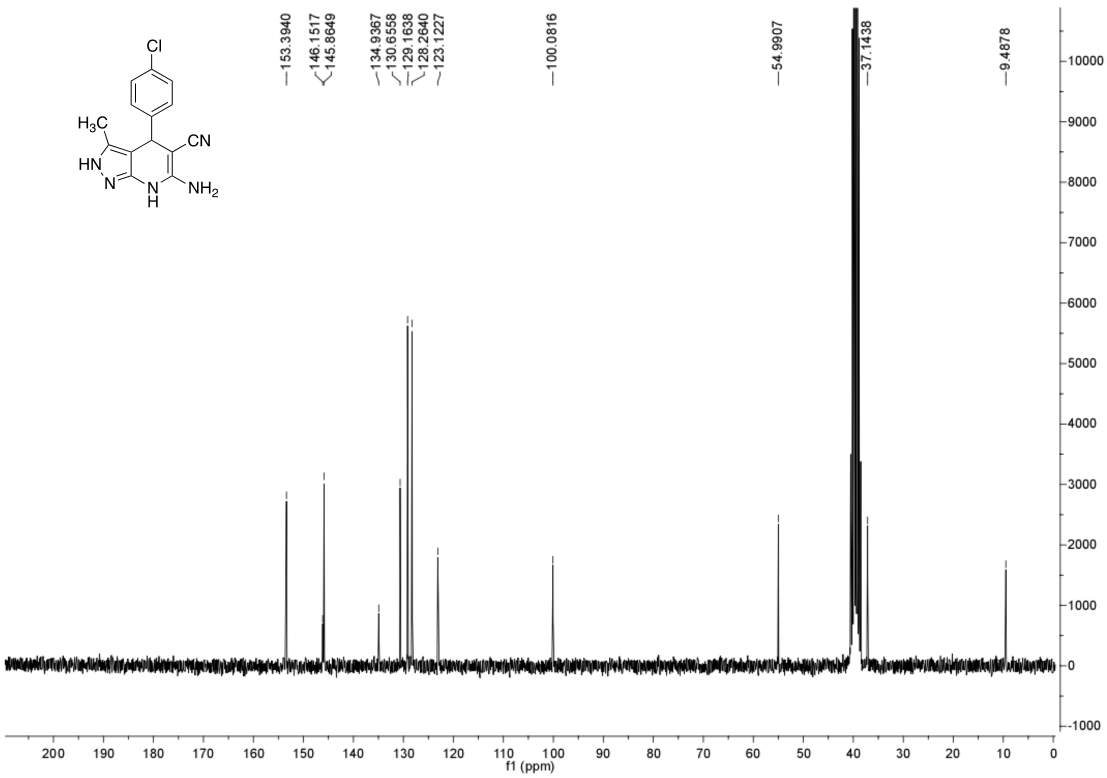

6-Amino-4-(2-bromophenyl)-3-methyl-4,7-dihydro-2*H*-pyrazolo[3,4-*b*]pyridine-5-carbonitrile 4f.

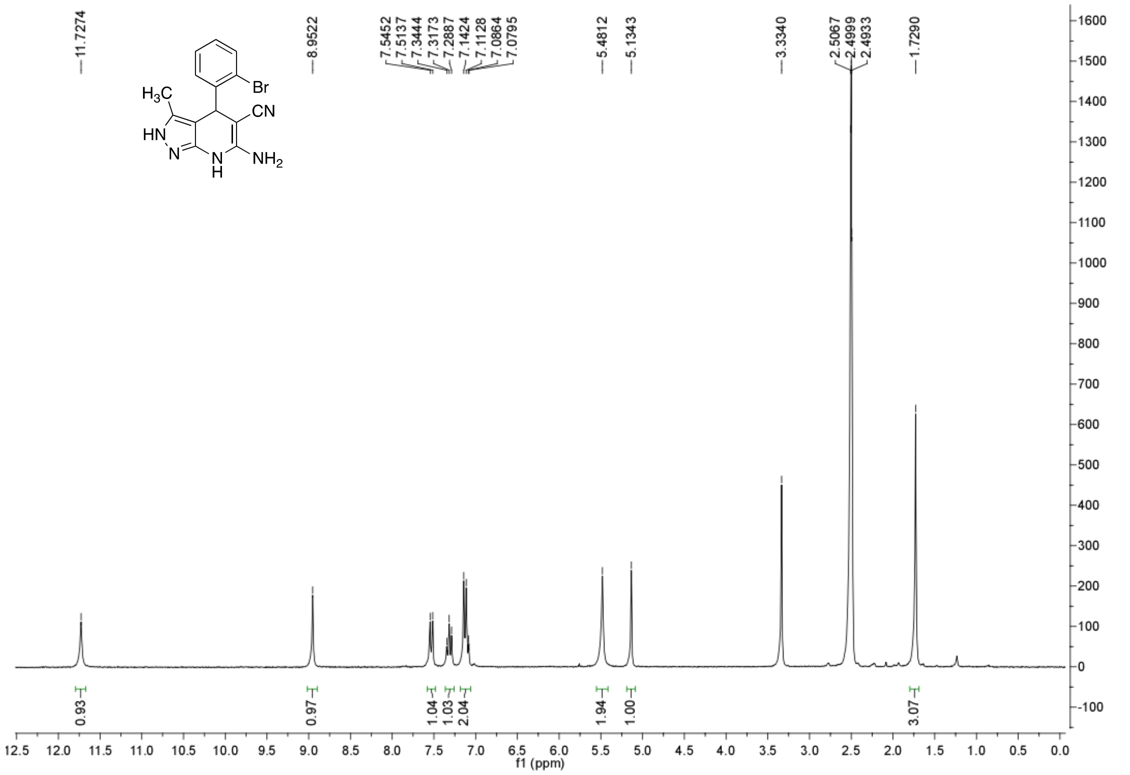

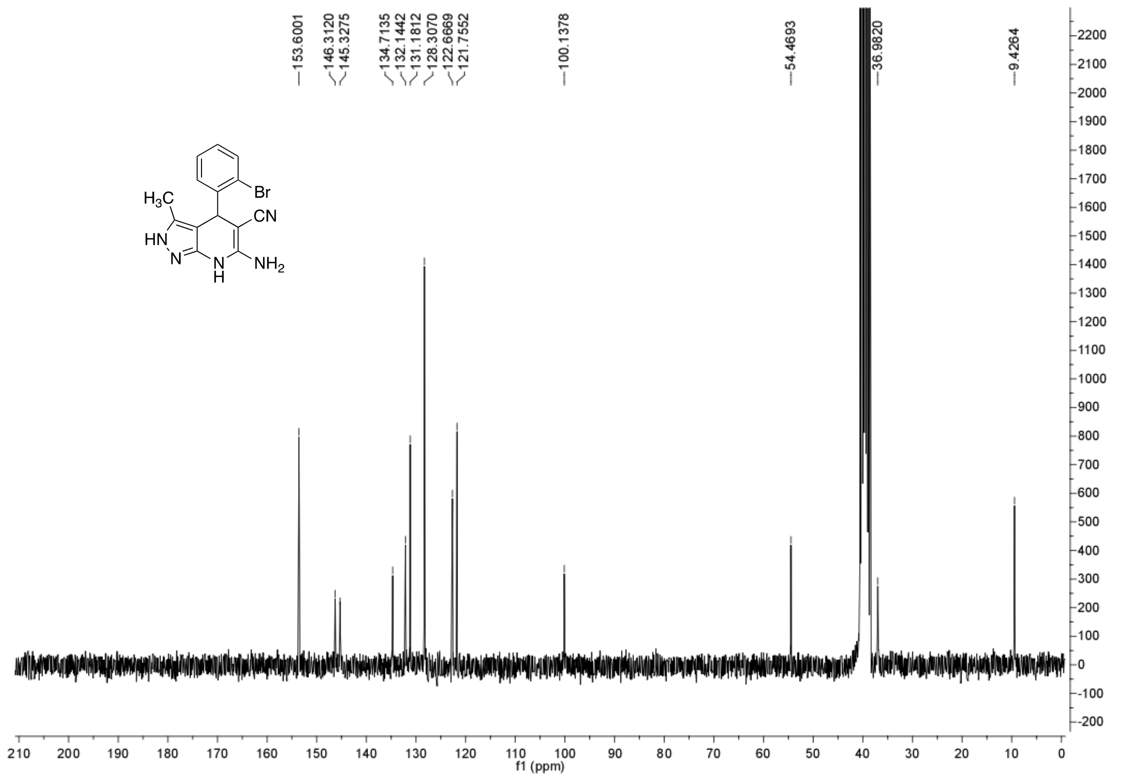

6-Amino-4-(4-bromophenyl)-3-methyl-4,7-dihydro-2*H*-pyrazolo[3,4-*b*]pyridine-5-carbonitrile 4g.

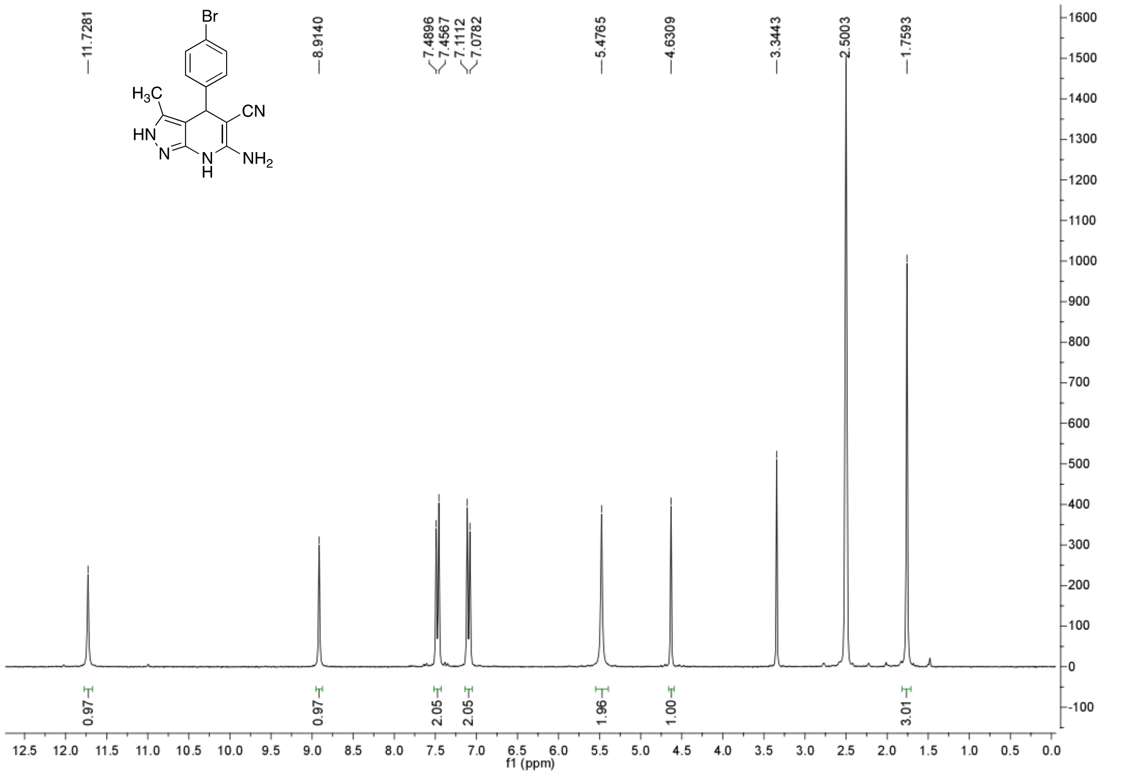

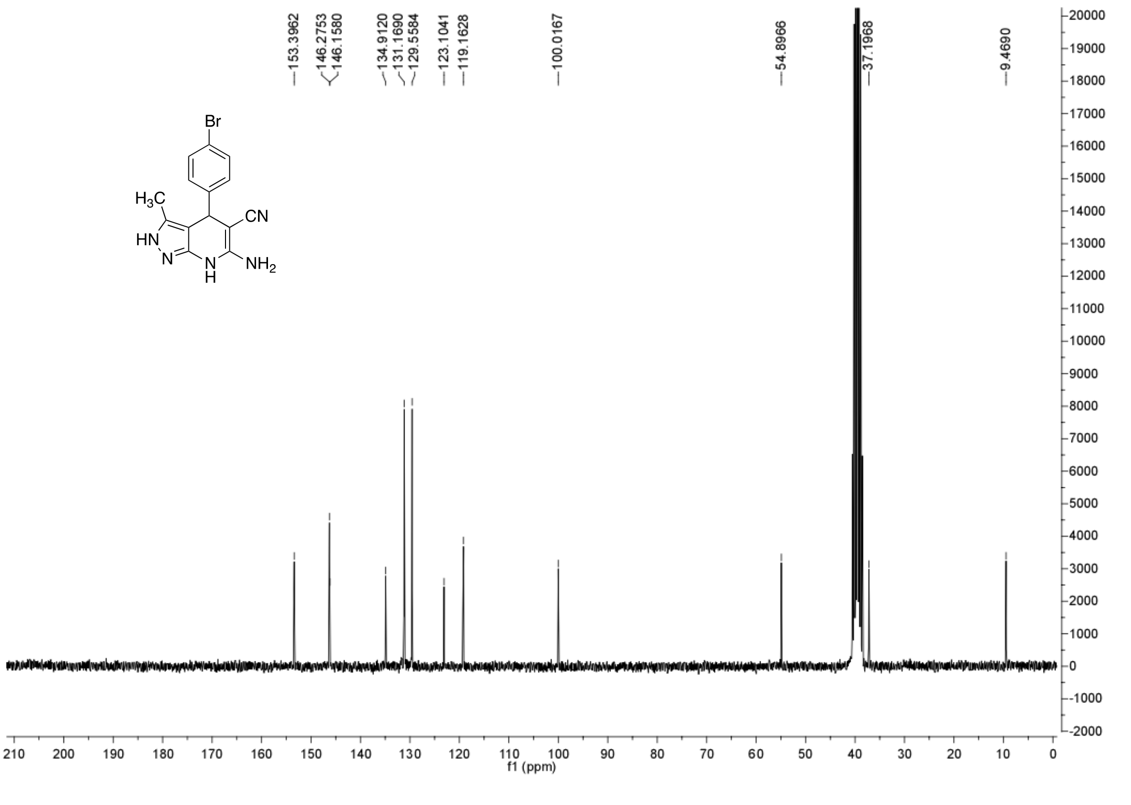

6-Amino-3-methyl-4-(3-nitrophenyl)-4,7-dihydro-2*H*-pyrazolo[3,4-*b*]pyridine-5-carbonitrile 4h.

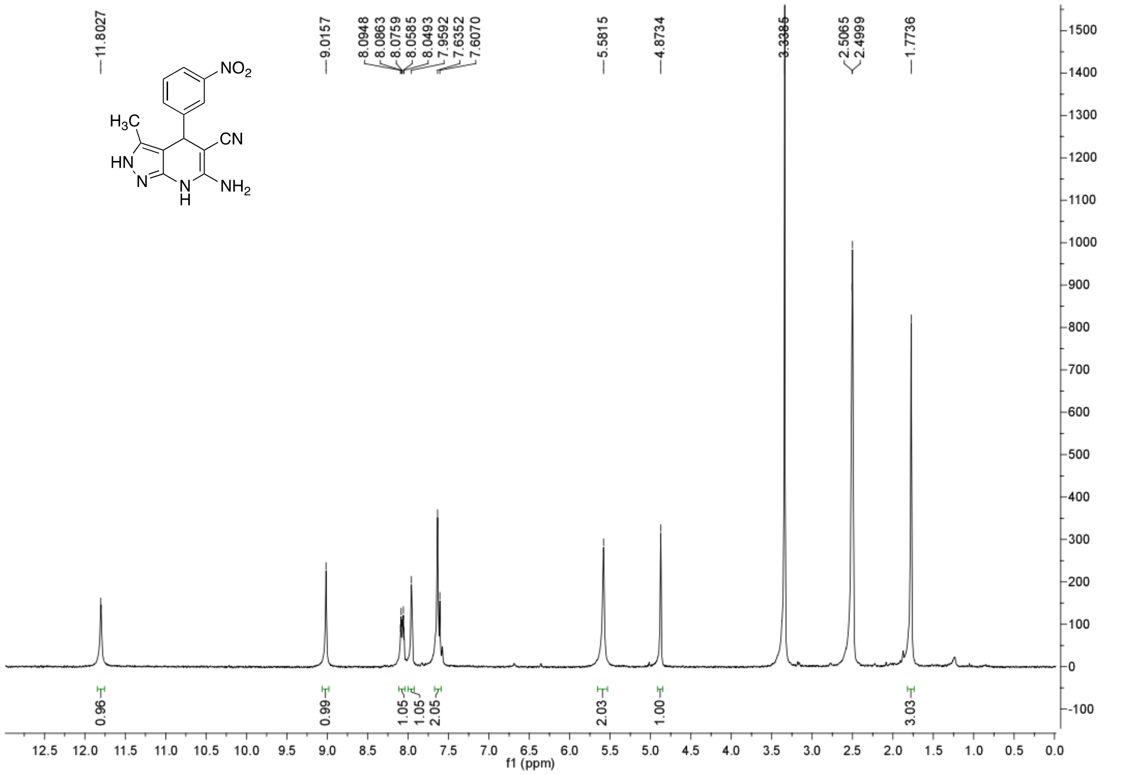

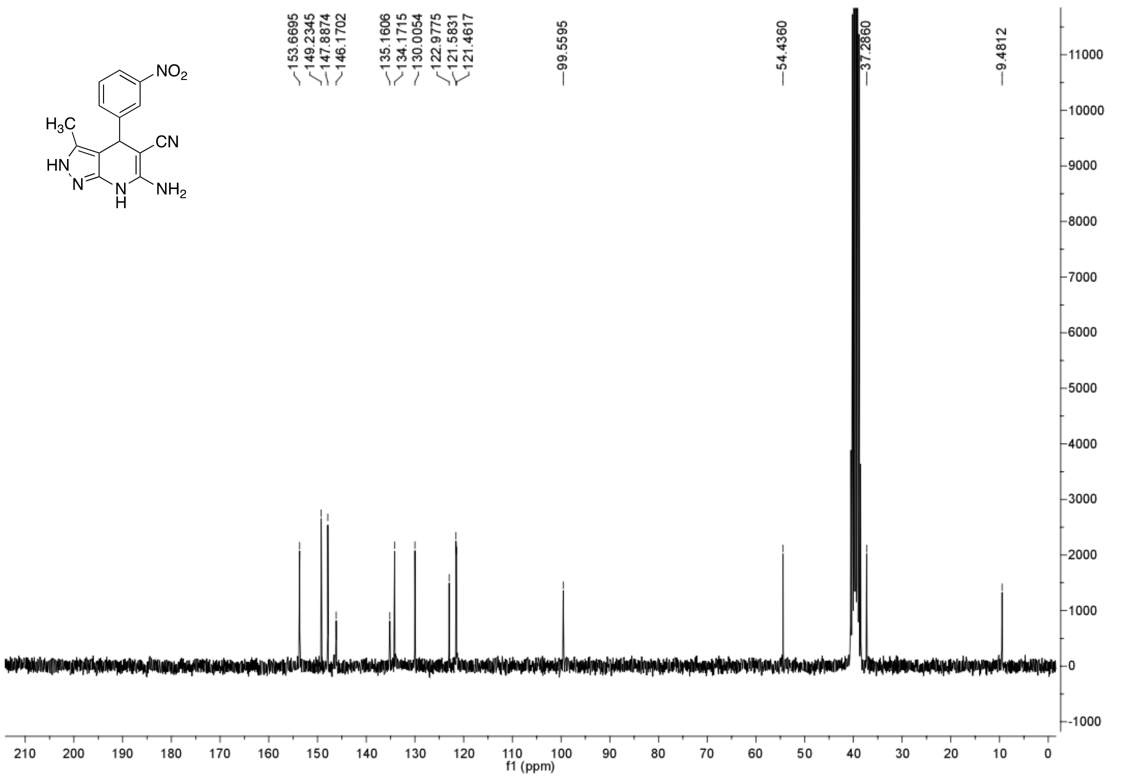

6-Amino-3-methyl-4-(4-nitrophenyl)-4,7-dihydro-2*H*-pyrazolo[3,4-*b*]pyridine-5-carbonitrile 4i.

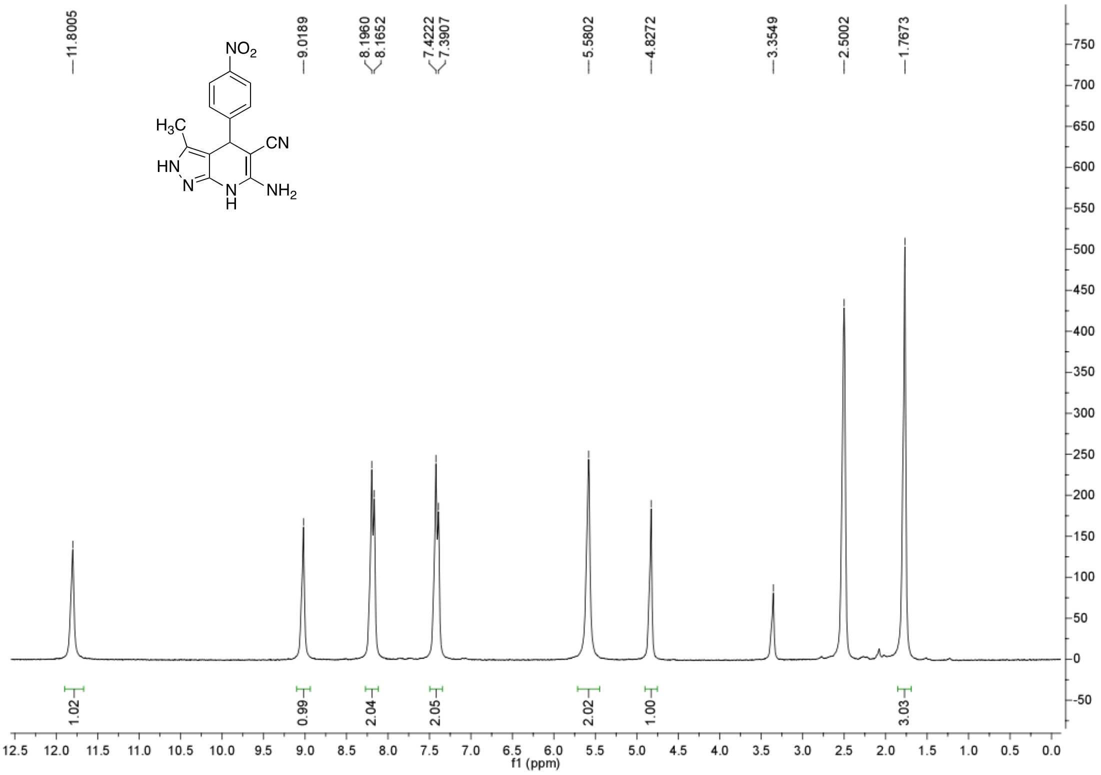

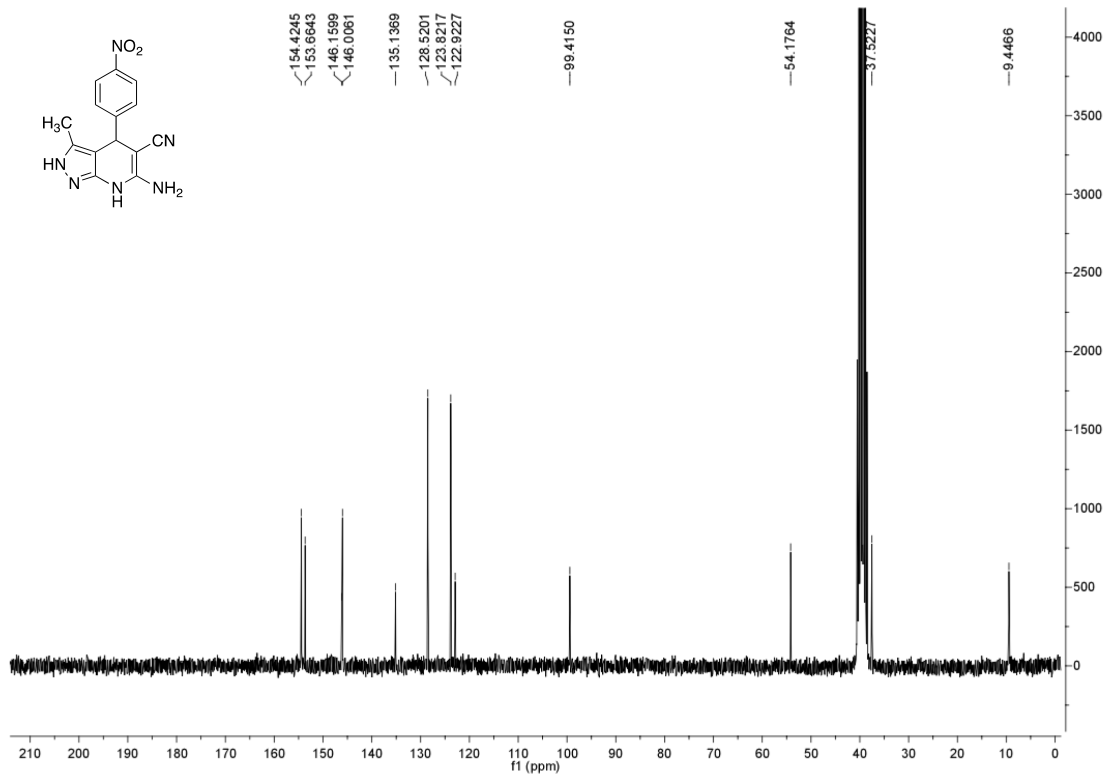

6-Amino-3-methyl-4-(thiophen-2-yl)-4,7-dihydro-2*H*-pyrazolo[3,4-*b*]pyridine-5-carbonitrile 4j.

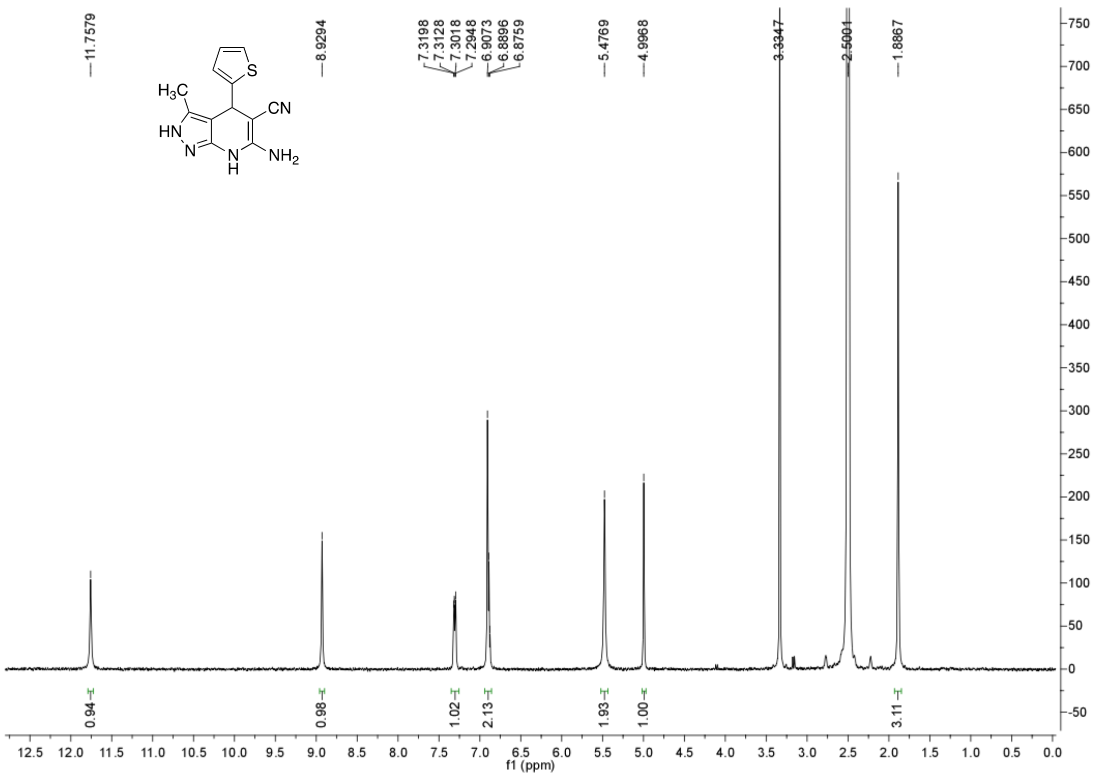

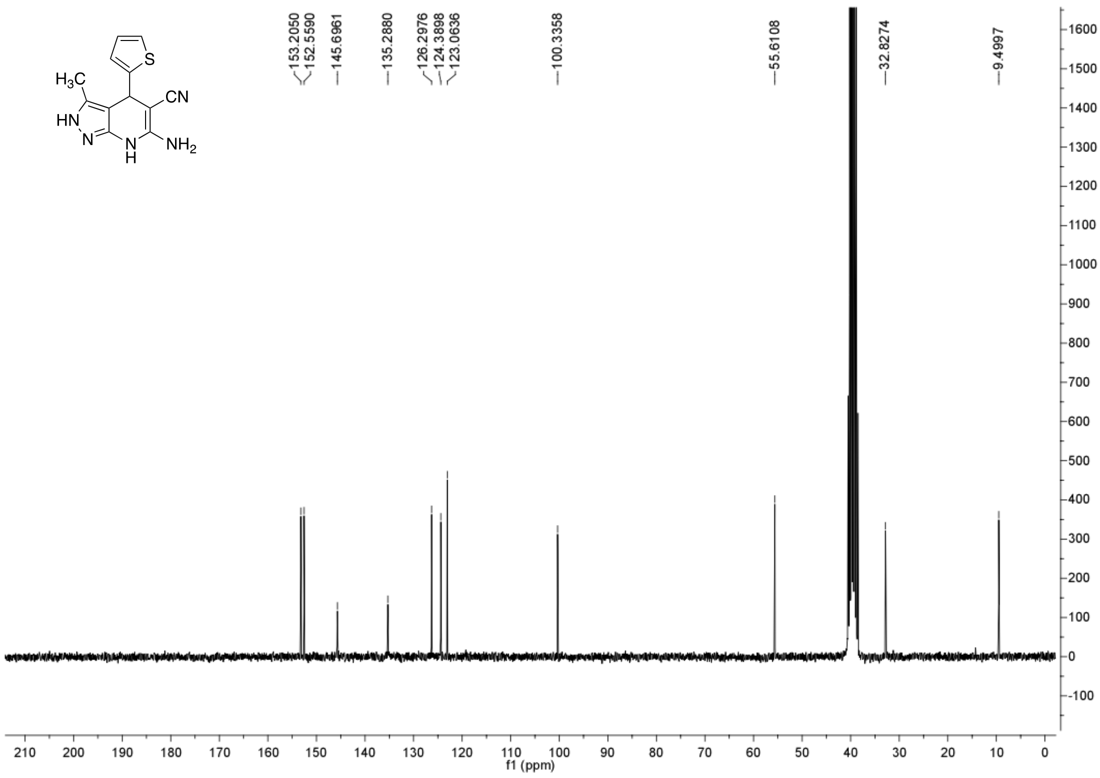

6-Amino-3-methyl-4-(pyridin-3-yl)-4,7-dihydro-2*H*-pyrazolo[3,4-*b*]pyridine-5-carbonitrile 4k.

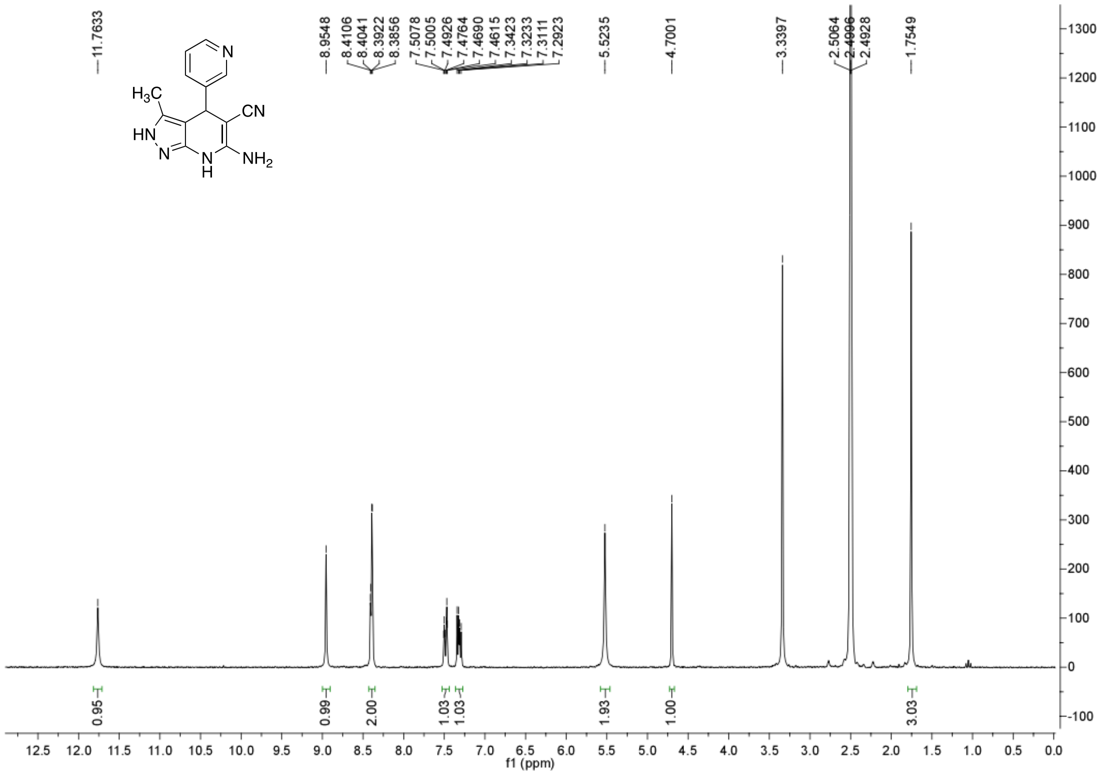

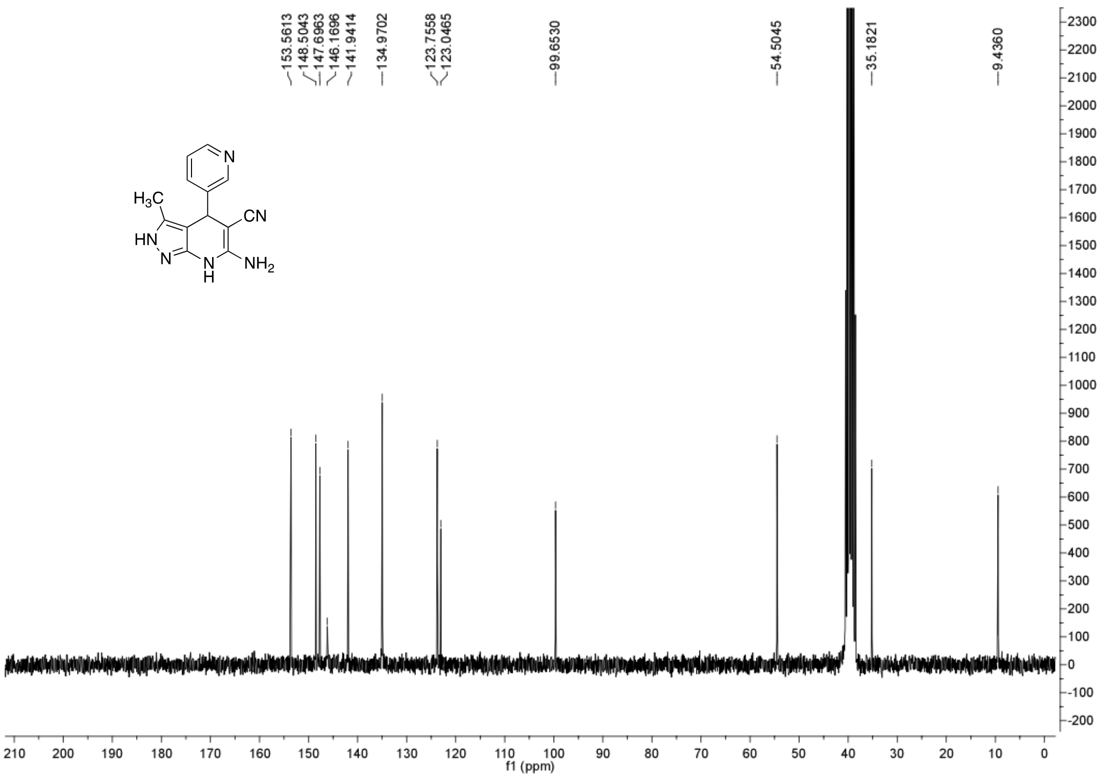

6-Amino-3-methyl-4-(pyridin-4-yl)-4,7-dihydro-2*H*-pyrazolo[3,4-*b*]pyridine-5-carbonitrile 4l.

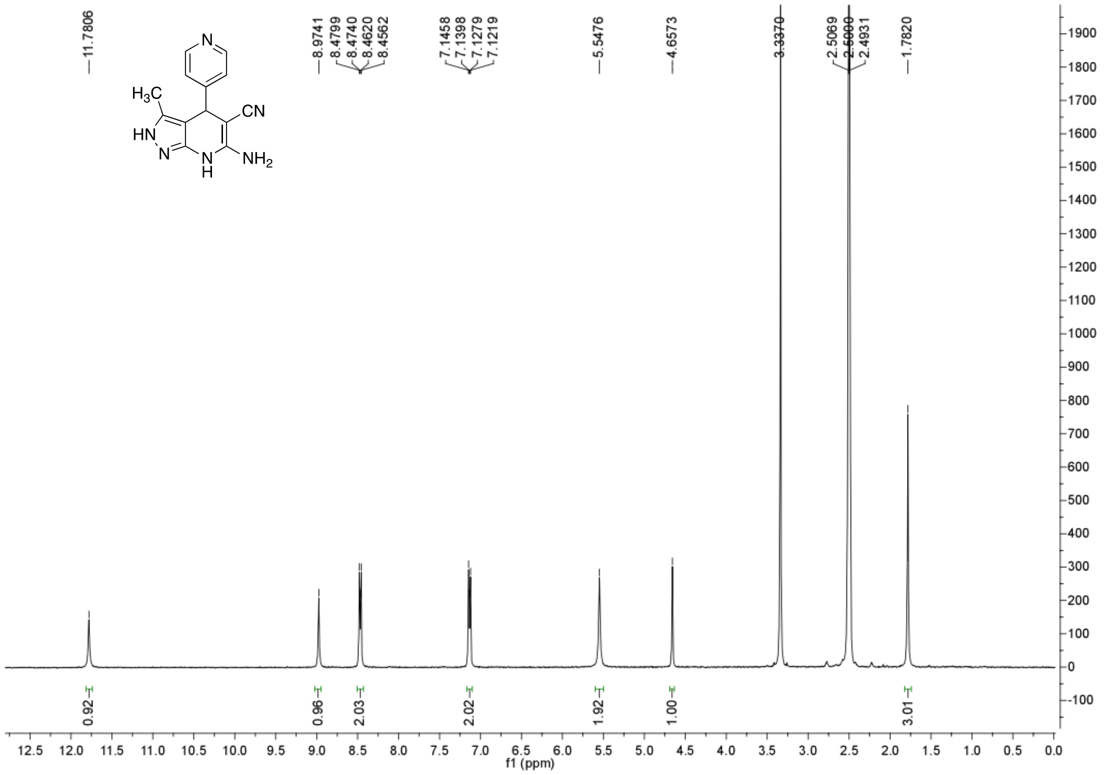

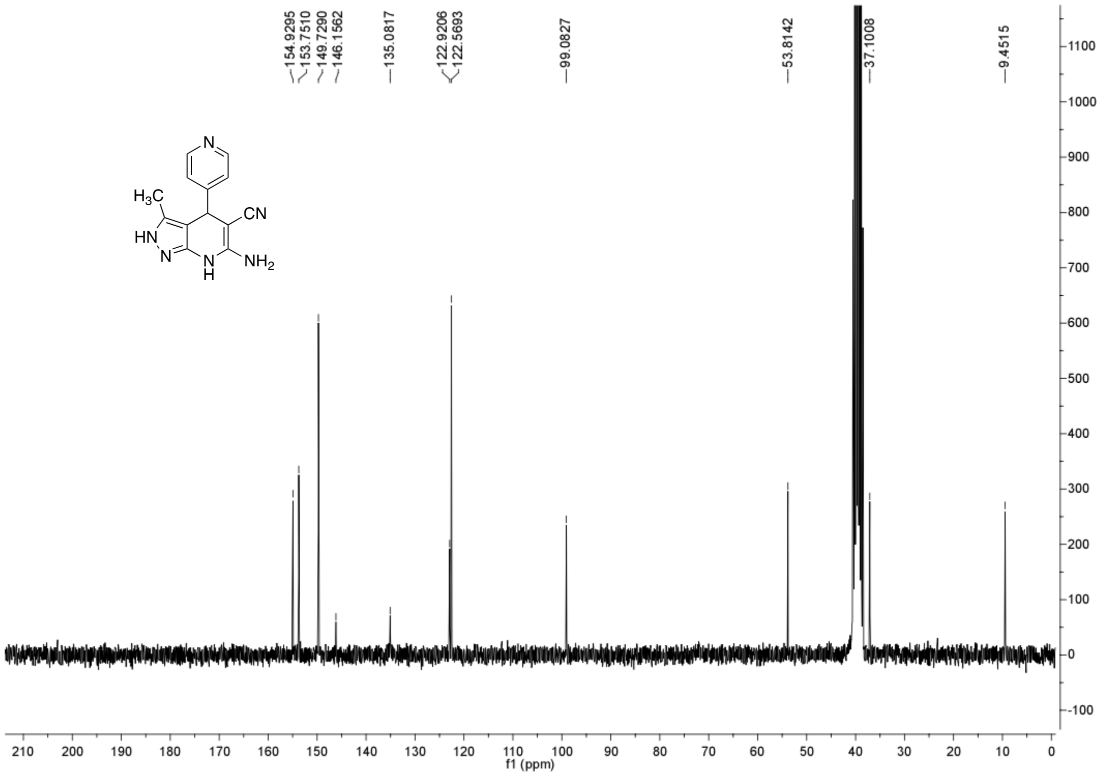

SI References

1. Gameiro, I.; Michalska, P.; Tenti, G.; Cores, A.; Buendia, I.; Rojo, A.I.; Georgakopoulos, N.D.; Hernandez-Guijo, J.M.; Teresa Ramos, M.; Wells, G., et al. Discovery of the first dual GSK3beta inhibitor/Nrf2 inducer. A new multitarget therapeutic strategy for Alzheimer's disease. *Sci Rep* **2017**, *7*, 45701, doi:10.1038/srep45701.
2. Cao, G.; Alessio, H.M.; Cutler, R.G. Oxygen-radical absorbance capacity assay for antioxidants. *Free Radic Biol Med* **1993**, *14*, 303-311, doi:10.1016/0891-5849(93)90027-r.
3. Ou, B.; Hampsch-Woodill, M.; Prior, R.L. Development and validation of an improved oxygen radical absorbance capacity assay using fluorescein as the fluorescent probe. *J Agric Food Chem* **2001**, *49*, 4619-4626, doi:10.1021/jf010586o.
4. Dudonne, S.; Vitrac, X.; Coutiere, P.; Woillez, M.; Merillon, J.M. Comparative study of antioxidant properties and total phenolic content of 30 plant extracts of industrial interest using DPPH, ABTS, FRAP, SOD, and ORAC assays. *J Agric Food Chem* **2009**, *57*, 1768-1774, doi:10.1021/jf803011r.
5. Baki, A.; Bielik, A.; Molnar, L.; Szendrei, G.; Keseru, G.M. A high throughput luminescent assay for glycogen synthase kinase-3beta inhibitors. *Assay Drug Dev Technol* **2007**, *5*, 75-83, doi:10.1089/adt.2006.029.
6. Cheng, Y.; Prusoff, W.H. Relationship between the inhibition constant (K1) and the concentration of inhibitor which causes 50 per cent inhibition (I50) of an enzymatic reaction. *Biochemical pharmacology* **1973**, *22*, 3099-3108.
7. Kozikowski, A.P.; Gaisina, I.N.; Yuan, H.; Petukhov, P.A.; Blond, S.Y.; Fedolak, A.; Caldarone, B.; McGonigle, P. Structure-based design leads to the identification of lithium mimetics that block mania-like effects in rodents. possible new GSK-3beta therapies for bipolar disorders. *Journal of the American Chemical Society* **2007**, *129*, 8328-8332, doi:10.1021/ja068969w.
8. Di, L.; Kerns, E.H.; Fan, K.; McConnell, O.J.; Carter, G.T. High throughput artificial membrane permeability assay for blood-brain barrier. *Eur J Med Chem* **2003**, *38*, 223-232, doi:10.1016/s0223-5234(03)00012-6.
9. Trott, O.; Olson, A.J. AutoDock Vina: improving the speed and accuracy of docking with a new scoring function, efficient optimization, and multithreading. *J Comput Chem* **2010**, *31*, 455-461, doi:10.1002/jcc.21334.
10. Tikhonov, D.B.; Zhorov, B.S. Structural model for dihydropyridine binding to L-type calcium channels. *J Biol Chem* **2009**, *284*, 19006-19017, doi:10.1074/jbc.M109.011296.
11. Denizot, F.; Lang, R. Rapid colorimetric assay for cell growth and survival. Modifications to the tetrazolium dye procedure giving improved sensitivity and reliability. *Journal of immunological methods* **1986**, *89*, 271-277.
